# 5-Iodotubercidin inhibits epithelial to mesenchymal transition by inhibiting IKK/NFκB-dependent gene expression

**DOI:** 10.1016/j.jbc.2026.113548

**Published:** 2026-09-12

**Authors:** Sayari Bhattacharya, Muskan Manjari, Vaishnavi Ganesh, Manoj B. Menon, Sonam Dhamija

**Affiliations:** 1 Kusuma School of Biological Sciences, Indian Institute of Technology Delhi, New Delhi, India; 2 CSIR-Institute of Genomics and Integrative Biology, New Delhi, India; 3 Faculty of Life Sciences and Biotechnology, South Asian University, New Delhi, India

**Keywords:** EMT, NFκB, 5-iodo-tubercidin, kinase inhibitor, prognostic signature

## Abstract

Epithelial to mesenchymal transition (EMT) is a process of trans-differentiation important for development, inflammation and cancer. Transforming Growth Factor-β (TGFβ) is a physiologically relevant inducer of EMT. We had recently characterized the adenosine analog, adenosine kinase inhibitor 5-Iodotubercidin (5-ITu), as a compound which preferentially sensitizes MK2-deficient cells to TNFα-induced, RIPK1-dependent cell death. Here we investigated the effect of 5-ITu on TGFβ-induced EMT. 5-ITu suppressed TGFβ-induced morphological changes and migration in A549 (lung cancer) and PANC1 (pancreatic cancer) cell lines. Consistent with these effects, there was significant suppression of EMT markers as indicated by qPCR, immunoblotting and immunofluorescence and confocal microscopy. Mechanistic investigations revealed that 5-ITu-mediated EMT suppression was independent of adenosine kinase inhibition and RIPK1 activation. 5-ITu suppressed NFκB activity in cells undergoing EMT and IKK inhibition phenocopied the effect of 5-ITu on EMT. The effect of 5-ITu on EMT was lost upon IκBα knockdown. Kinase assays revealed IKKβ as a potential direct target of 5-ITu. We identified a TGFβ-associated, NFκB-dependent gene signature consisting of four genes, that are differentially regulated upon 5-ITu treatment. Interestingly, this four gene signature could predict survival in lung and pancreatic cancer. The identification of this role for the multitarget kinase inhibitor 5-ITu in NFκB activity-dependent EMT, in addition to RIPK1-dependent necroptosis has potential implications in anticancer strategies.

Epithelial to Mesenchymal Transition (EMT) is a trans-differentiation process where epithelial cells lose their inherent features like cell-cell contacts, cell polarity and gain mesenchymal phenotypes (1). EMT is a critical player in development and in the process of cancer progression and metastasis. TGFβ is the best characterized cytokine known to induce EMT in a wide variety of mammalian epithelial cells and cell line models (2, 3). Downstream to TGFβ-receptors, SMAD2/3/4 transcription factors regulate key players that orchestrate the EMT program. The canonical EMT signaling includes the repression of epithelial genes like E-cadherin (*CDH1*) and the upregulation of mesenchymal genes like Vimentin (*VIM*), N-cadherin (*CDH2*), and fibronectin (*FN1*). SMAD signaling mediates the transcriptional reprogramming by inducing the expression of key transcriptional factors such as Snail (SNAI1), Slug (SNAI2), ZEB1, and ZEB2, which in turn suppress CDH1 expression (4). Concurrently, they promote the transcription of mesenchymal genes conferring migratory and invasive properties. In addition, other non-canonical signaling pathways activated by TGFβ, and involved in EMT include MAPK pathways, PI3K/Akt pathway, NFκB and Rho-like GTPase signaling (3, 5).

5-Iodotubercidin (5-ITu) is a nucleoside analog and has been reported as a potent adenosine kinase inhibitor (6). *In vitro* and *in vivo* studies have established strong anti-cancer potential of 5-ITu (7). Adenosine kinase inhibition by 5-ITu was shown to suppress SARS-CoV-2 replication (8). Despite its discovery as an adenosine kinase inhibitor, 5-ITu was later characterized as a protein kinase inhibitor (9). The earliest characterized protein kinase targets of 5-ITu include CK1, CK2, and ERK2 (10, 11). In addition, DYRK1A inhibition by 5-ITu was shown to modulate β-cell proliferation and function (12). Another well-established target of 5-ITu is the histone-H3 kinase Haspin/GSG2 (13, 14, 15).

In our recent study, we identified 5-ITu as a small molecule modulator of RIPK1-dependent death. In a small-molecule inhibitor screen, 5-ITu was shown to specifically sensitize MK2-deficient cells to TNF-induced necroptosis (16). TNFα-induced cell death is modulated mainly by two checkpoints involving IKK and MK2-mediated RIPK1 phosphorylation, which prevents RIPK1 autophosphorylation and subsequent cytotoxic response (17, 18, 19, 20, 21). Mechanistically, 5-ITu-mediated inhibition of IKK-dependent RIPK1 phosphorylation was found to facilitate RIPK1 autophosphorylation and predominant necroptotic response in cells lacking MK2 activity (16).

In the present work, we investigated the effect of 5-ITu on EMT. 5-ITu strongly suppressed TGFβ-induced EMT as evident from EMT marker expression, morphological analyses and cell migration. We present evidence that 5-ITu inhibits NFκB signaling by directly targeting the IKKs, independent of adenosine kinase inhibition. In contrast to the effect of 5-ITu on TNFα-induced cytotoxicity, the suppression of TGFβ-induced EMT was independent of RIPK1 activity. Moreover, we identified an NFκB-dependent gene signature, sensitive to 5-ITu, and modulated by TGFβ. Interestingly, TCGA (The Cancer Genome Atlas) data analyses revealed that this 4-gene signature is associated with poor survival in lung and pancreatic cancer patients.

## Results

### 5-ITu inhibits TGFβ-induced epithelial to mesenchymal transition

Our previous studies have identified 5-ITu as an inhibitor of TNF-induced survival signaling and 5-ITu was shown to facilitate TNF-induced cell death synergistically with p38/MK2 inactivation (16). To understand the effect of 5-ITu on other physiologically and pathologically relevant events, we focused on TGFβ-induced epithelial to mesenchymal transition (EMT). Co-treatment with 5-ITu suppressed the TGFβ-induced morphological changes in A549 (lung adenocarcinoma) and PANC1 (pancreatic ductal carcinoma) cell lines (Figs. 1*A* and S1*A*). Consistent with the EMT-like morphological changes, immunofluorescence analyses revealed upregulation of filamentous actin (phalloidin staining) and redistribution of epithelial marker E-cadherin (CDH1) in TGFβ-treated A549 cells. These effects were also suppressed by treatment with 5-ITu (Fig. 1*B*). To quantitatively assess the effect of 5-ITu on TGFβ-induced EMT in A549 cells, we monitored the expression of epithelial (E-cadherin/*CDH1*) and mesenchymal (N-cadherin/*CDH2*, *ZEB1*, Snail/*SNAI1*) marker genes by real-time qPCR. 5-ITu co-treatment significantly suppressed the TGFβ-induced upregulation of mesenchymal transcripts and downregulation of epithelial marker *CDH1* (Fig. 1*C*). While the effects of 5-ITu on TGFβ-regulated marker gene expression were comparable to those of the TGFβ-receptor kinase inhibitor SB431542, 5-ITu additionally suppressed the basal expression levels of EMT transcription factors *SNAI1* and *ZEB1*. Similar results were also observed in TGFβ-treated PANC1 cells (Fig. S1*B*). Consistent with the effect at the RNA level, TGFβ-induced changes in CDH1, CDH2 and SNAI1 protein levels were also reversed by 5-ITu treatment (Figs. 1*D* and S1*C*). EMT is associated with enhanced capacity for cell migration, often evaluated by scratch wound-healing assays (22). TGFβ-treatment increased the wound closure capacity of both A549 and PANC1 cells when assayed by a quantitative scratch wound-healing assay. 5-ITu dose-dependently inhibited wound closure in both cell lines (Fig. 1*E*). Consistent with the effects on mesenchymal marker expression, 5-ITu-mediated suppression of migration was stronger than that of SB431542. These findings clearly establish 5-ITu as an inhibitor of EMT.

**Figure 1:**
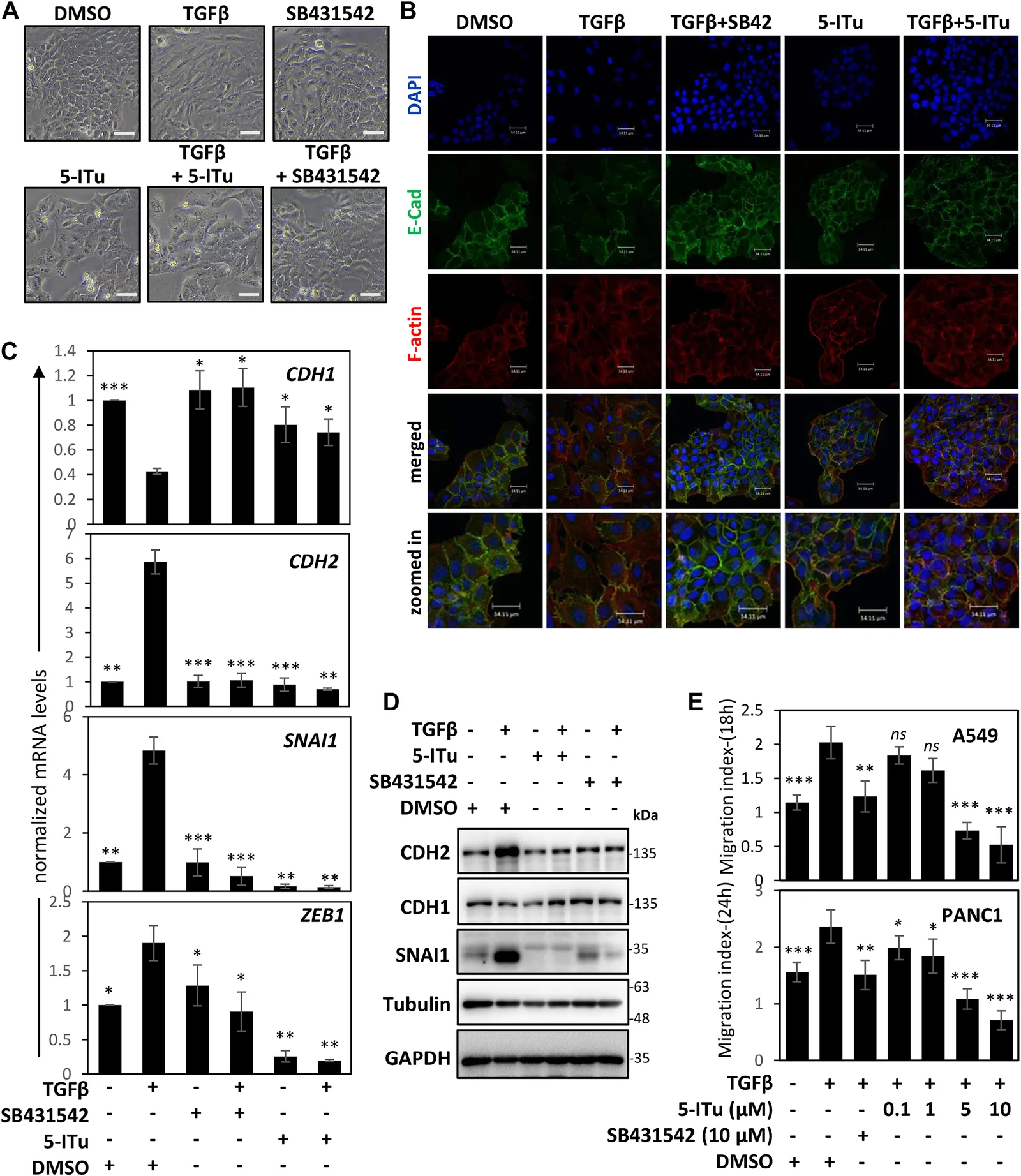
**5-Iodotubercidin (5-ITu) inhibits TGFβ-induced EMT.***A*, A549 cells were treated as indicated for 24 h and images were acquired using a phase-contrast 10x objective. Scale bars indicate 50 μm. *B*, cells seeded on cover slips were treated for 24 h as indicated. After fixation, cells were stained for F-actin (phalloidin), DNA (DAPI) and E-cadherin/CDH1 (E-cad) and were imaged by confocal microscopy. *C* and *D*, A549 Cells were stimulated with TGFβ (5 ng/ml) in combination with indicated inhibitors-SB431542 (10 μM), 5-ITu (5 μM) or solvent control (DMSO) and EMT marker expression was monitored by qPCR (n = 3, mean ± SD for three independent experiments) (*C*) and immunoblotting (*D*). *E*, A549 (*upper panel*) and (PANC1) cells were subjected to a quantitative scratch wound healing assay for 18 h and 24 h respectively and migration indices were plotted (n ≥ 4, mean ± SD). (∗ denotes *p*-value ≤ 0.05, ∗∗ denotes *p*-value ≤ 0.01, ∗∗∗ denotes *p*-value ≤ 0.001).

### Adenosine kinase inhibition does not phenocopy the effect of 5-ITu on EMT

5-ITu is a nucleoside analog and is best characterized as an adenosine kinase inhibitor (23). Adenosine kinase inhibition can lead to the accumulation of extracellular adenosine driving purinergic signaling, with a potential effect on EMT. To understand whether 5-ITu-mediated suppression is an aftereffect of adenosine kinase inhibition, we monitored the effect of a structurally unrelated adenosine kinase inhibitor ABT-702 on TGFβ-induced EMT (24). Unlike 5-ITu, ABT-702 treatment did not significantly alter the TGFβ-induced morphological changes in A549 and PANC1 cells (Fig. 2*A*). There was a minor but significant effect of ABT-702 on TGFβ-induced expression of SNAI1 in A549 cells, but similar effects were not observed on the expression of other marker genes tested (Figs. 2, *B* and *D* and S2*A*). Comparable effects were observed in PANC1 cells (Figs. 2, *C* and *E* and S2*B*), with no inhibition of TGFβ-induced EMT markers seen with ABT-702. While there was a significant inhibitory effect of ABT-702 on TGFβ-induced migration in A549 cells, the effect was not observed in PANC1 cells (Fig. 2, *F* and *G*). Moreover, the effect of 5 μM 5-ITu was significantly stronger than the effect shown by ABT-702 in either cell lines. These results indicate that the EMT-inhibitory effect of 5-ITu is probably independent of its effect on adenosine kinase activity.

**Figure 2:**
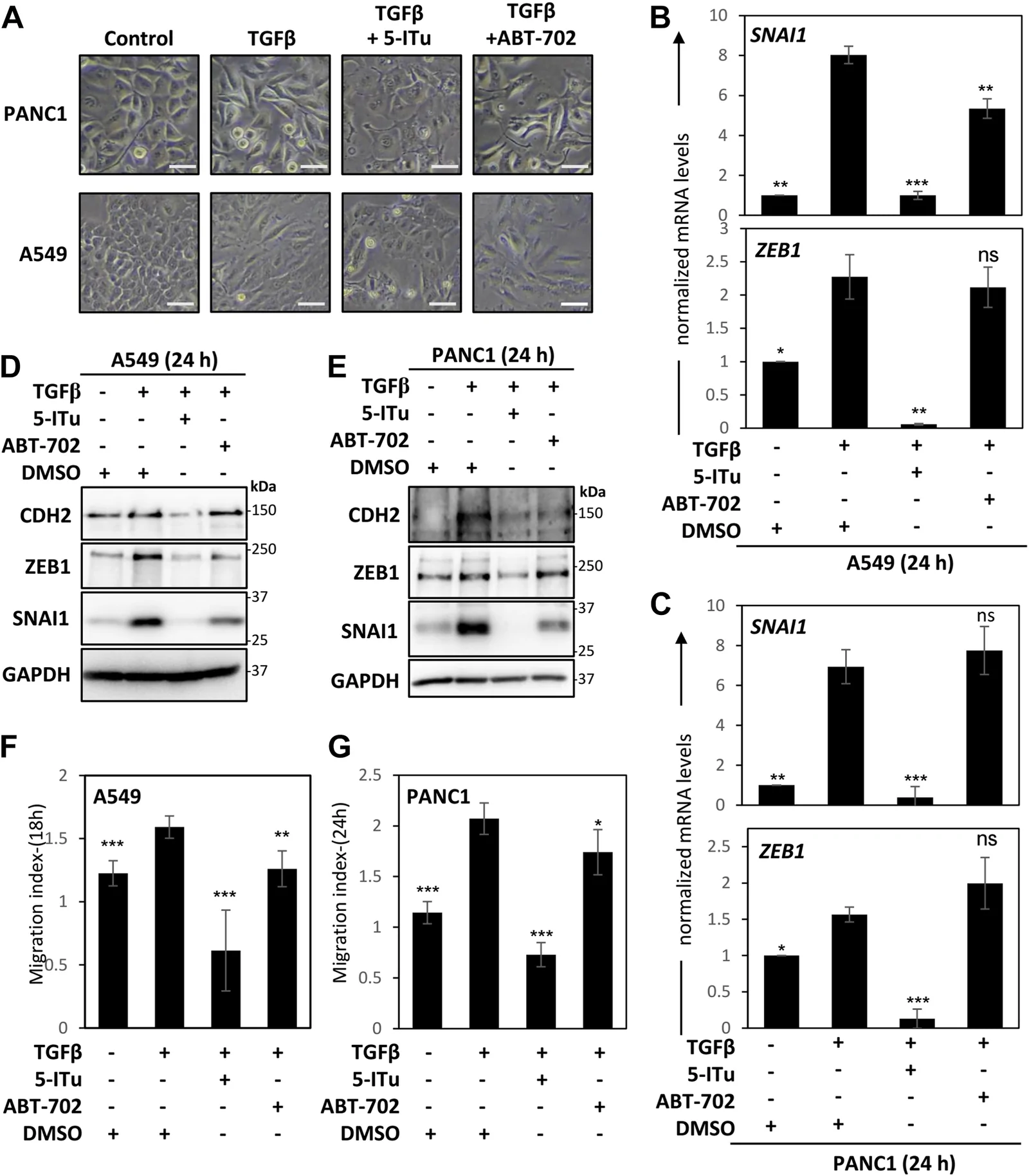
**Adenosine kinase inhibition does not phenocopy the effect of 5-ITu on EMT.***A*, A549 cells were treated as indicated for 24 h and images were acquired using a phase-contrast 10x objective. Scale bars indicate 50 μm. *B–E*, A549 (*B* & *D*) or PANC1 (*C* & *E*) cells were stimulated with TGFβ in combination with indicated inhibitors (5-ITu: 5 μM, ABT702: 10 μM) or solvent control (DMSO) and EMT marker expression was monitored by qPCR (n = 3, mean ± SD plotted) (*B* & *C*) and immunoblotting (*D* & *E*). *F* and *G*, A549 (*F*) or PANC1 (*G*) cells were subjected to a quantitative scratch wound healing assay in the presence of stimulants/inhibitors as indicated and migration indices were plotted (n ≥ 5, mean ± SD). (∗ denotes *p*-value ≤ 0.05, ∗∗ denotes *p*-value ≤ 0.01, ∗∗∗ denotes *p*-value ≤ 0.001).

### The effect of 5-ITu on EMT is independent of RIPK1-mediated cytotoxicity

Our previous work had established an adenosine-kinase independent role for 5-ITu in facilitating TNFα and LPS-induced apoptosis/necroptosis through the hyperactivation of RIPK1. To monitor whether a similar cytotoxic response is responsible for the effect of 5-ITu on TGFβ-induced EMT, we performed cell viability assays comparing the effect of 5-ITu on TGFβ and TNFα treatment. When cell viability was assayed using WST-8 assays, 5-ITu displayed a dose-dependent decrease in cell viability at 48 h. However, there was no effect of TGFβ co-treatment on viability. On the other hand, co-treatment with TNFα induced further loss of A549 cell viability (Fig. 3*A*). We also monitored the presence of cell death in TGFβ and TNFα-treated cells by use of Sytox-green assay which measures the presence of secondary necrotic cells. Consistent with the viability assay results, TNFα, and not TGFβ co-treatment with 5-ITu led to a significant increase in cell death as indicated by enhanced Sytox-green fluorescence (Fig. 3*B*). Interestingly, TGFβ-TNFα co-treatment, an established and more effective EMT stimulus behaved more like TNFα-treated cells in both viability and cytotoxicity assays (Fig. 3, *A* and *B*). Moreover, the adenosine kinase inhibitor ABT-702 did not affect cell viability in the conditions tested (Fig. 3, *A* and *B*). Similar results were observed in PANC1 cells (Fig. 3*C*). These data indicate that the effect of 5-ITu on TGFβ signaling is not linked to RIPK1-mediated cytotoxicity. Flow-cytometry–based cell cycle analyses revealed an effect of 5-ITu on G2/M arrest (Figs. 3*D* and S3), which may explain the reduction in the proportion of viable cells observed in the WST-8 assays. Enhanced cytotoxicity upon TNFα and 5-ITu co-treatment is associated with RIPK1 activation-dependent ripoptosome/necrosome assembly (16). Consistent with this, TNFα/5-ITu-mediated cytotoxic response was sensitive to the RIPK1 inhibitor necroststain-1 (Nec-1) (Fig. 3*E*). However, co-treatment with RIPK1 inhibitors Nec-1 or GSK481 did not have an impact on the effect of 5-ITu on TGFβ-induced EMT as indicated by the expression of the EMT markers *SNAI1* and *CDH2* (Fig. 3*F*). These findings clearly establish that 5-ITu interferes with TGFβ-induced EMT, independent of RIPK1 activation and apoptosis/necroptosis.

**Figure 3:**
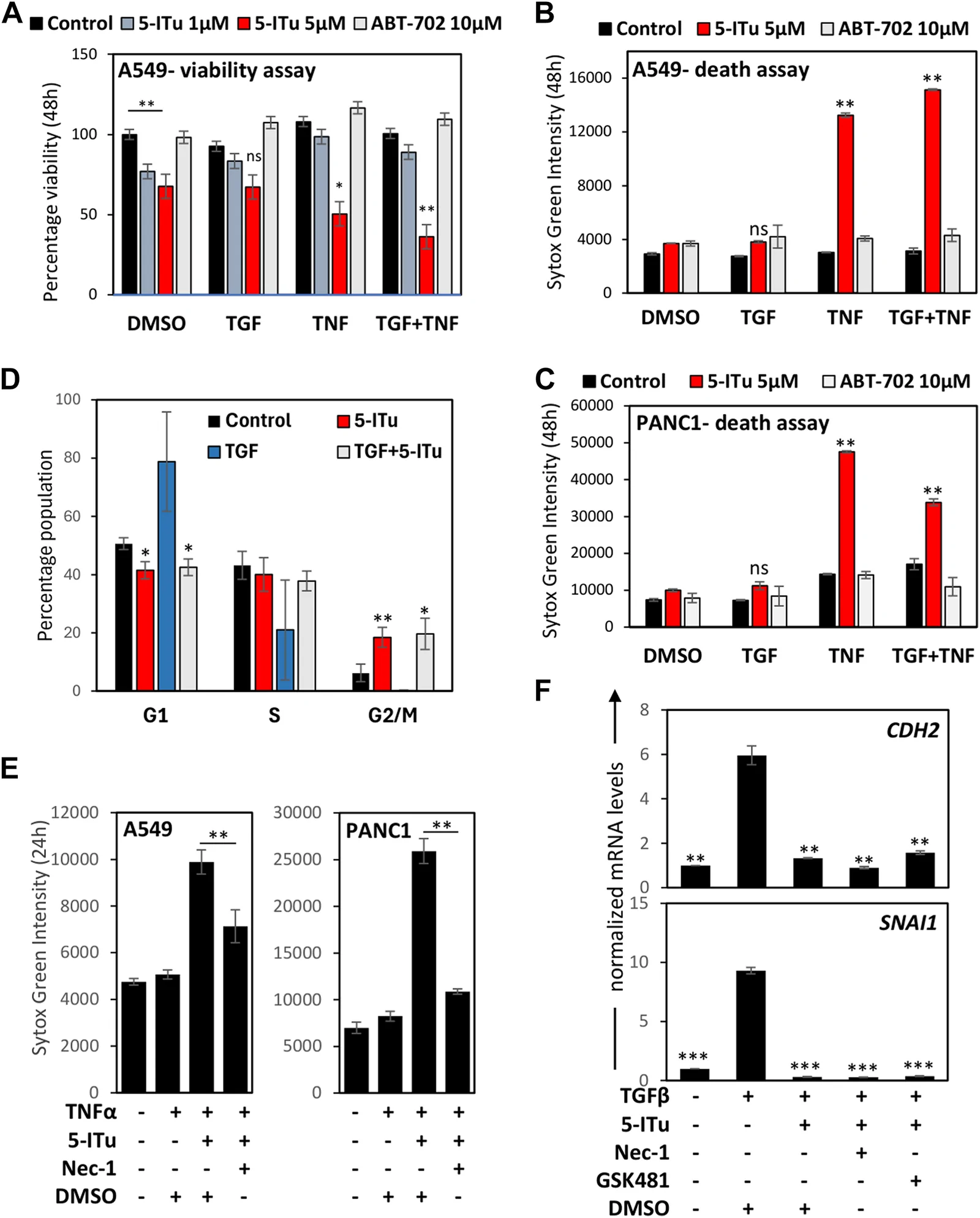
**5-ITu mediated suppression of EMT is independent of RIPK1-dependent cytotoxicity.***A*, A549 cells were seeded in a 96 well plate and treated with TGFβ, TNFα in combination with indicated inhibitors or solvent control (DMSO) for 48 h. Cell viability was measured using WST-8. *B* and *C*, A549 (*B*) and PANC1 cells (*C*) were treated with combinations indicated for 48 h and cell death was measured and are indicated as Sytox *green* intensities. *D*, Flow cytometry analysis of cell cycle distribution in A549 cells. Cells were treated as indicated for 24 h, cell cycle phase distribution was calculated and presented (n = 3, mean ± SD plotted). *E*, A549 (*left*) and PANC1 cells (*right*) were treated with combinations indicated for 24 h and cell death was measured. *F*, A549 cells were stimulated with TGFβ in combination with indicated inhibitors or solvent control (DMSO) and EMT marker expression was monitored by qPCR (mean ± SD plotted). The concentrations of inhibitors used, unless indicated are 5-ITu: 5 μM, Nec-1: 50 μM and GSK481: 5 μM. (∗ denotes *p*-value ≤ 0.05, ∗∗ denotes *p*-value ≤ 0.01, ∗∗∗ denotes *p*-value ≤ 0.001).

### The effect of 5-ITu on EMT is associated with the inhibition of IKK/NFκB signaling

To further delineate the mechanisms of 5-ITu-mediated suppression of EMT, we investigated the NFκB activation status in the cells undergoing TGFβ-induced EMT. Cells treated for 24 h with TGFβ showed significant amounts of IκBα protein and phosphorylated NFκB/p65. However, IκBα and phospho-p65 levels were not significantly affected compared to control cells (Figs. 4*A* and S4*A*). Interestingly, 5-ITu treatment led to a strong suppression of p65 phosphorylation, indicating inhibition of IKK activity and suppression of NFκB-dependent transcription activity. Interestingly, TGFβ receptor-kinase inhibition by SB431542 was associated with enhanced p65 phosphorylation and IκBα-levels. On the other hand, 5-ITu did not interfere with TGFβ receptor kinase activity as monitored by SMAD2/3 phosphorylation (Fig. S4, *B* and *C*). We then asked the question whether inhibition of NFκB signaling by IKK1/2 inhibitors will phenocopy the effect of 5-ITu on EMT. The IKK inhibitor BMS345541 suppressed the morphological changes induced by TGFβ in A549 and PANC1 cells (Fig. 4*B*). Consistent with the effect on the EMT-related morphological changes, IKK inhibition also suppressed the EMT-associated gene expression changes at RNA and protein levels (Figs. 4, *C–E* and S4, *D–F*), albeit with reduced efficacy compared to 5-ITu. To conclusively prove a role for 5-ITu in the suppression of NFκB-dependent transcription, we performed reporter assays using an NFκB-response element-driven reporter construct. As expected, TNFα treatment led to a strong upregulation of NFκB-dependent luciferase activity, while TGFβ induced the reporter activity to a lesser extent (Fig. 4*F*). Consistent with the effects observed on p65 phosphorylation, 5-ITu treatment strongly suppressed TNFα and TGFβ-induced reporter activity, similar to the IKKα/β (IKK1/2) inhibitor BMS345541 (25). NFκB signaling is known to regulate EMT markers both directly and indirectly (26, 27, 28, 29). Analysis of ChIP-Seq data from the Gene Transcription Regulation Database (https://gtrd.biouml.org/) revealed that at least some of the marker gene promoters are directly bound to NFκB family transcription factors (Fig. S4*G*). To further explore the potency of 5-ITu in inhibiting NFκB-dependent transcription, we performed dose-response experiments. Interestingly, 5-ITu efficiently suppressed both TNF- and TGF-induced NFκB reporter activity at sub-micromolar concentrations with IC_50_ values of 207 nM and 297 nM, respectively (Fig. 5, *A* and *B*).

**Figure 4:**
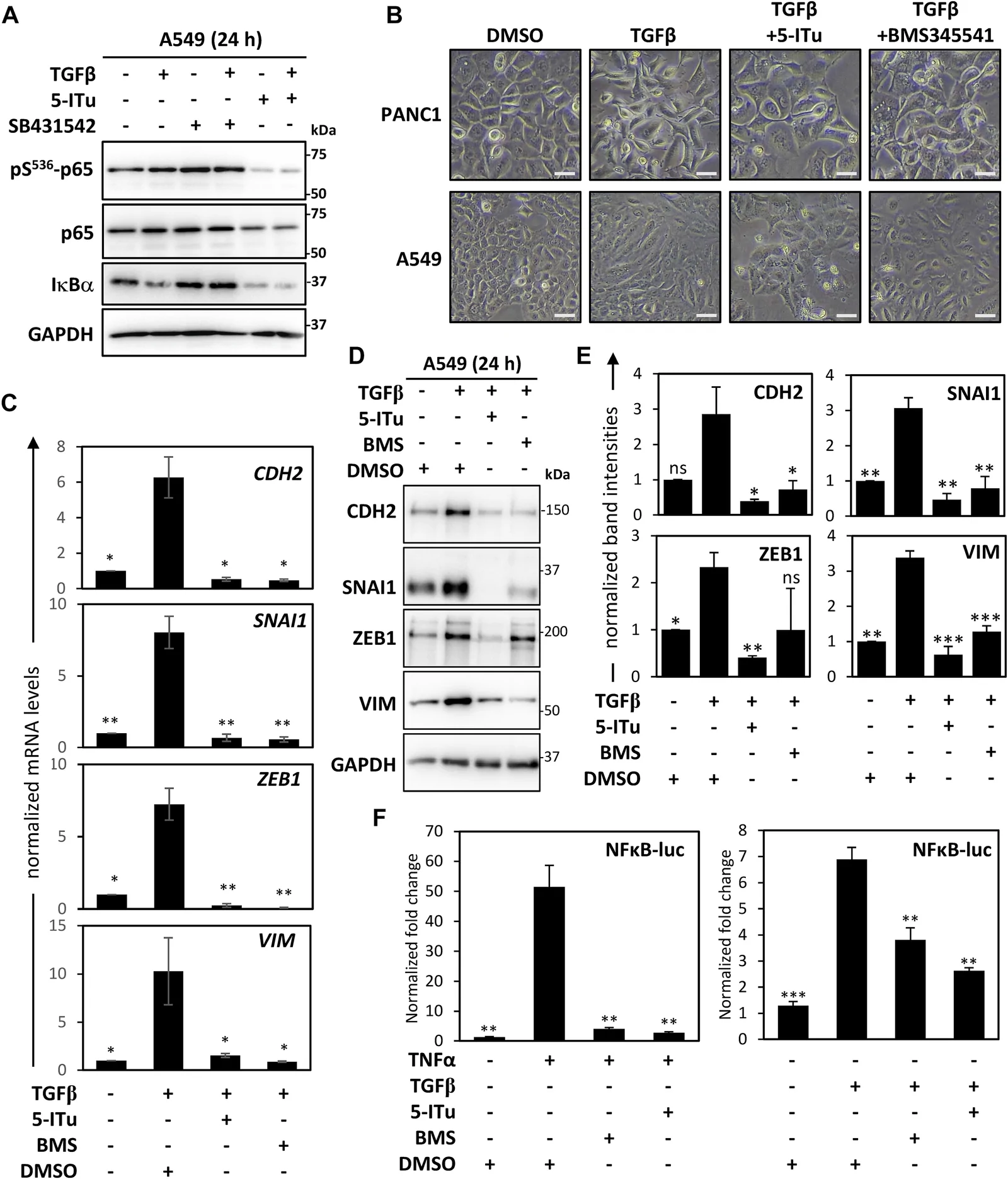
**5-ITu inhibits EMT by suppressing NFκB-dependent transcription.***A*, A549 cells were treated with TGFβ in combination with the inhibitors 5-ITu and SB42 for 24 h and lysates were probed with pS^536^-p65, total p65 and IκBα to show the effect of 5-ITu on the NFκB pathway. *B*, PANC1 and A549 cells were treated as indicated for 24 h and images were acquired using a phase-contrast 10x objective. Scale bars indicate 50 μm. *C–E*, A549 cells were stimulated with TGFβ in combination with indicated inhibitors or solvent control (DMSO) and EMT marker expression was monitored by qPCR (mean ± SD plotted) (*C*) and immunoblotting (*D*). Immunoblot band intensities of EMT markers normalized to GAPDH are shown (n = 3) (*E*). *F*, HEK293T cells transfected with luciferase reporter plasmid and Renilla luciferase were treated as indicated for 6 h. Luminescence was measured in microplate reader and calculated normalized relative luciferase activity. The concentrations of inhibitors used are 5-ITu: 5 μM, BMS: 5 μM and SB431542: 10 μM. (∗ denotes *p*-value ≤ 0.05, ∗∗ denotes *p*-value ≤ 0.01, ∗∗∗ denotes *p*-value ≤ 0.001).

**Figure 5:**
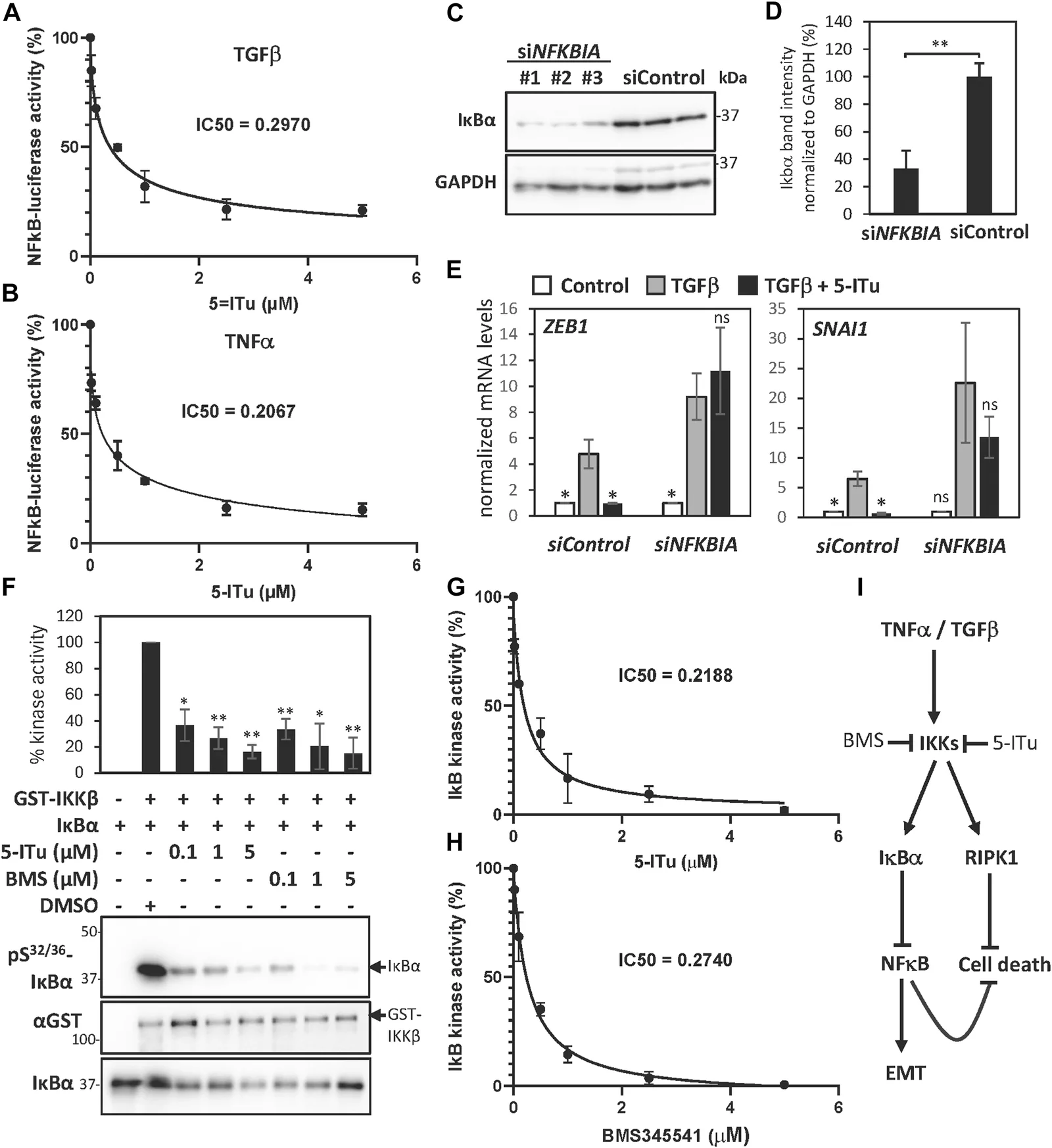
**IKK is a direct target of 5-ITu relevant to the suppression of EMT.***A* and *B*, NFκB reporter assays were performed in HEK293T cells treated with various doses of 5-ITu (0.02, 0.1, 0.5, 1, 2.5 and 5 μM) or solvent control (DMSO), in the presence of TGFβ (*A*) or TNFα (*B*) as activators of signaling. The normalized luciferase activity values (%) were plotted and IC_50_ values determined using Graphpad Prism software. *C* and *D*, A549 cells were transfected with control siRNA or three independent siNFKBIA siRNAs and the IκBα protein levels after 48 h were evaluated by immunoblotting (*C*). The percentage knockdown calculated by densitometry normalized to loading control (GAPDH) is shown (*D*). *E*, siRNA transfected cells were treated for 24 h as indicated with TGFβ (5 ng/ml) and solvent control (DMSO) or 5-ITu (5 μM) and EMT marker expression was evaluated by RT-qPCR. *F*. *In vitro* kinase assay performed by incubating the kinase IKKβ and the substrate IκBα along with three different concentrations of 5-ITu and BMS345541 as indicated for 30 min. SDS gel was run for the reaction mixes and probed with pS32/S36- IκBα *versus* total IκBα and with GST. *G* and *H*, Kinase assays were performed as in *panelF* with different doses (0.02, 0.1, 0.5, 1, 2.5 and 5 μM) of 5-ITu (*G*) or BMS345541 (*H*) and the normalized band intensities were plotted, to calculate IC_50_ values using Graphpad Prism software. *I*, a schematic summarizing the role of IKK/NFκB signaling in EMT and the interference by 5-ITu, comparing it to the known effect on RIPK1-dependent cell death. (∗ denotes *p*-value ≤ 0.05, ∗∗ denotes *p*-value ≤ 0.01, ∗∗∗ denotes *p*-value ≤ 0.001).

The primary role of IKKs in NFκB signaling is to facilitate phosphorylation-dependent degradation of IκBα. We hypothesized that loss of IκBα would render IKKs nonessential for TGFβ-induced NFκB activation and downstream EMT. This will also nullify the effect of 5-ITu on EMT. For this, we resorted to siRNA-mediated depletion of IκBα. Three independent siRNAs against the human IκBα gene (*NFKBIA*) were used to efficiently knock down the protein in A549 cells (Fig. 5, *C* and *D*). As expected, IκBα knockdown led to the upregulation of TGF-induced EMT marker expression compared to control siRNA-transfected cells (Fig. 5*E*). Interestingly, while TGFβ-induced expression of *SNAI1* and *ZEB1* in control siRNA-transfected cells was suppressed upon 5-ITu treatment, 5-ITu did not significantly downregulate EMT marker expression in IκBα knockdown cells (Fig. 5*E*). This conclusively proves that suppression of IκBα degradation is the predominant mechanism by which 5-ITu suppresses EMT.

### IKKβ is a direct protein kinase target of 5-ITu,as demonstrated by *in vitro* kinase assays

Earlier efforts to identify the direct target of 5-ITu relevant to its effect on IKK signaling were unsuccessful (16). Considering that the effect of 5-ITu on IKK/NFκB signaling is conserved downstream to TGFβ as well as TNFα receptors, we hypothesized that 5-ITu could be directly binding IKK and inhibiting IKK catalytic activity. To test this hypothesis, we performed *in vitro* kinase assay for IKKβ using recombinant IκBα as substrate. BMS345541, a well-characterized IKKβ inhibitor was used as a positive control (25). Interestingly, 5-ITu dose-dependently inhibited IκBα phosphorylation with an efficiency comparable to that of BMS345541 (Fig. 5*F*). To further confirm that 5-ITu is a potent inhibitor of IKK, we performed dose-response experiments and compared the IC50 values of 5-ITu and BMS345541. *In vitro* kinase assays for IκBα kinase activity revealed an IC_50_ of 219 nM for 5-ITu (Fig. 5*G*) compared to 274 nM for BMS345541 (Fig. 5*H*) These findings indicate that 5-ITu-mediated inhibition of TGFβ-mediated EMT is a consequence of direct IKK inhibition by 5-ITu, resulting in the suppression of NFκB signaling (Fig. 5*I*).

### A 5-ITu-sensitive NFκB-dependent gene signature relevant to EMT and cancer

TGFβ-mediated EMT is mainly regulated by the SMAD2/3-dependent signaling pathways altering the expression of EMT-related transcription factors and cadherin family proteins. Previous studies have established a SMAD-independent role for NFκB signaling in the regulation of EMT (5, 30). This is consistent with the effect of IKK inhibitor BMS345541 on TGFβ-induced EMT and expression of EMT-related genes (Fig. 4, *B*–*E*). To further confirm that the effect of 5-ITu on EMT is mediated by the suppression of NFκB-dependent gene transcription, we compared the gene expression signatures unique to TGFβ signaling and that of TNF-NFκB pathway. The gene sets for TGFβ and TNFα/NFκB pathways were obtained from the Molecular Signatures Database (www.gsea-msigdb.org) (31, 32) and consisted of 54 and 200 genes respectively. There were ten genes common between the two datasets indicating these are potential NFκB-dependent transcripts downstream to TGFβ signaling (Fig. 6*A*). Interestingly, expression of this 10 gene signature was associated with overall survival in lung cancer (Fig. 6*B*). Similar effects were also observed in case of pancreatic cancer (Fig. S5*A*). We then asked the question whether these genes are sensitive to 5-ITu treatment. While four of the genes (*SMAD3*, *SERPINE1*, *TGIF1*, and *PMEPA1*) displayed TGFβ-induced upregulation as well as suppression by 5-ITu treatment (Fig. 6*C*), three genes (*BMP2*, *KLF10* and *IFNGR2*) were not expressed to detectable levels in A549 cells, independent of TGFβ stimulation. While *JUNB* displayed a reverse tendency, the expression changes were not statistically significant, and two genes (*ID2* and *PPP1R15A*) did not display significant alterations in response to TGFβ or 5-ITu treatment (Fig. 6*D*). We also monitored the effect of 5-ITu on the expression of *XIAP*, a target and regulator of NFκB pathway as well as EMT (33). Moreover, 5-ITu treatment also inhibited TGFβ-induced *XIAP* expression (Fig. 6*E*). Interestingly, a four gene signature defined by (*i*) TGFβ/TNFα dependence and (*ii*) sensitivity to 5-ITu was enough to predict overall survival in lung (Fig. 6*F*) and pancreatic cancer (Fig. S5*B*). Moreover, higher expression of these four genes was associated with poorer disease-free survival (Fig. 6, *G* and *H*), revealing the prognostic significance of the 5-ITu–sensitive gene signature.

**Figure 6:**
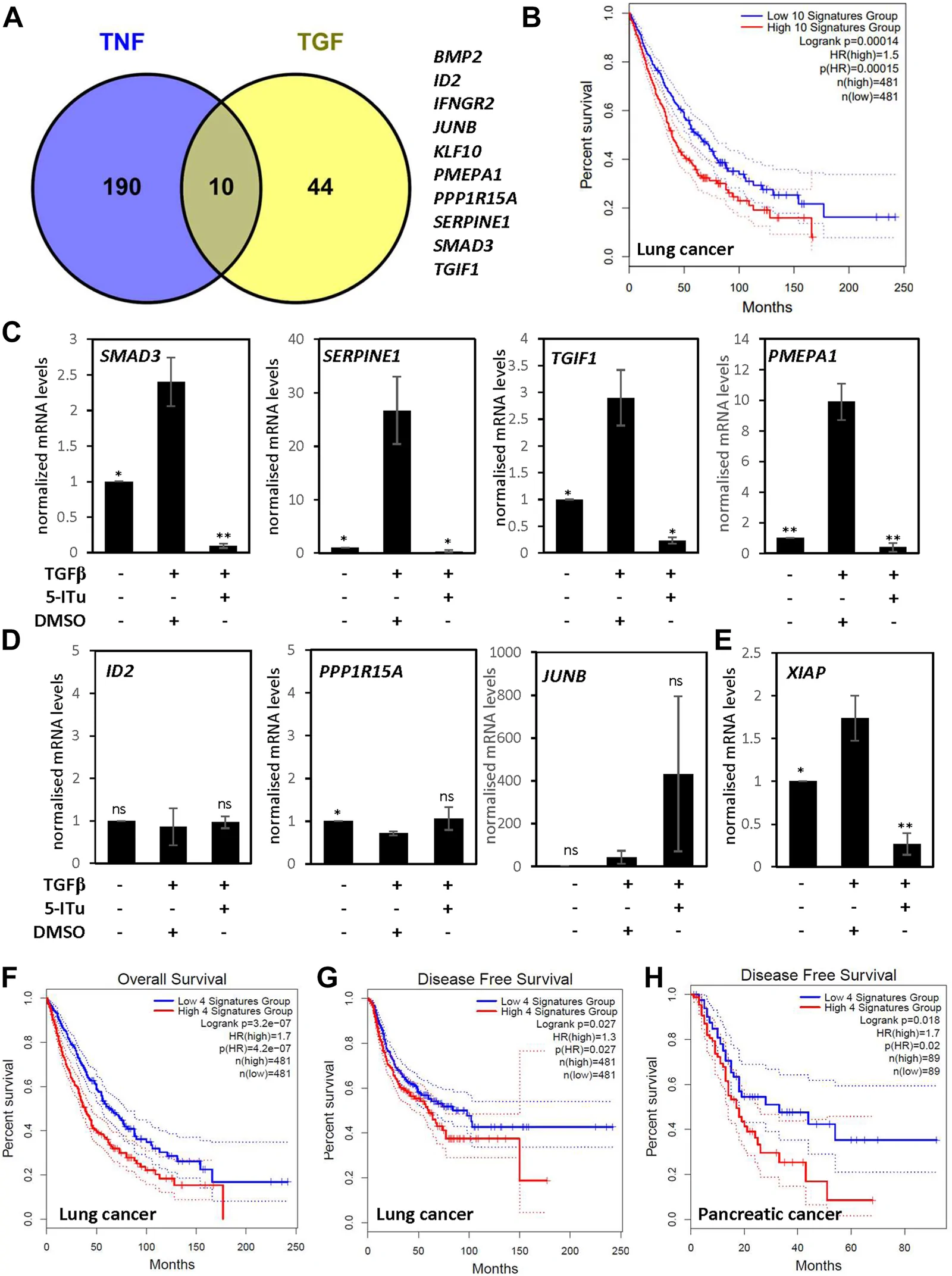
**AnNFκB-dependent, 5-ITu-sensitive gene signature predicts disease-free survival in cancer.***A*, a Venn diagram depicting the common genes in the hallmark genes of TNF/NFκB and TGFβ signaling. The 10 common genes present in both gene sets are shown in the *right panel*. *B*, the 10-gene signature predicts overall survival in lung cancer. *C–E*, A549 cells were treated with TGFβ alone or in combination with 5-ITu (5 μM) for 24 h and expression of the indicated transcripts were analyzed by qRT-PCR (∗ denotes *p*-value ≤ 0.05, ∗∗ denotes *p*-value ≤ 0.01, ∗∗∗ denotes *p*-value ≤ 0.001). *F*, Kaplan-Meier plot generated from the GEPIA2 server shows the prediction of overall survival in lung cancer by the 5-ITu-sensitive 4-gene signature. *G* and *H*, Kaplan-Meier plots show the prediction of disease-free survival in lung cancer (*G*) and pancreatic cancer (*H*) by the 5-ITu-sensitive 4-gene signature. Median expression value used for high/low expression determination in all survival plots and other statistical parameters are shown in the plots.

## Discussion

We had earlier identified 5-ITu in a screen for small-molecule inhibitors that sensitize MK2-KO MEFs to TNF-induced necroptosis (16). 5-ITu was shown to facilitate RIPK1 activation by compromising an IKK1/2-mediated checkpoint on RIPK1 activation. Here we present evidence for 5-ITu as a strong inhibitor of type-II EMT induced by TGFβ. Consistent with the effect on TNFα and TLR4 signaling, the effect of 5-ITu on IKK signaling was also conserved in case of TGFβ-induced EMT. Unlike in the case of TNFα/LPS, 5-ITu did not enhance cytotoxicity in combination with TGFβ. However, 5-ITu treatment significantly affected viable cell numbers and cell cycle progression independent of TGFβ. This is in line with the role of 5-ITu as a nucleoside analog and potential chemotherapeutic agent. As established in the literature, TGFβ is a weak activator of NFκB signaling compared to TNFα and co-treatment with TNFα enhances TGFβ-induced EMT, by activating strong NFκB signaling (34). This is evident from the NFκB-luciferase reporter assays which showed almost 10-fold difference in the induction of reporter activity between TNFα and TGFβ (Fig. 4*E*). Despite weak activation of NFκB pathway by TGFβ, NFκB-dependent transcription is key to the EMT program. Consistent with this, IKK2 inhibition by specific inhibitor BMS345541 suppressed EMT like 5-ITu, and 5-ITu treatment downregulated NFκB/p65 phosphorylation concomitant with inhibition of TGFβ-induced EMT. Interestingly, in immunoblots of samples from cells treated with TGFβ-receptor kinase inhibitor SB431542, we observed the accumulation of IκBα and enhanced phospho-p65 levels (35). Such crosstalk between long-term inactivation of TGFβ receptor activity and hyperactivation of IKK signaling has not been reported previously and warrants further investigation.

EMT is essential for embryonic development and is involved in cancer progression and metastasis (36, 37). EMT-associated transcription factors like SNAI1, SNAI2 and ZEB1 are implicated in several forms of cancer (38, 39, 40, 41). While the role of NFκB signaling in cancer has been investigated more in the context of inflammation induced carcinogenesis, survival signaling and suppression of apoptotic pathways (42), NFκB-dependent transcription also play key roles in EMT, metastasis and development of more aggressive and invasive cancers (28). Interestingly, a recent study used the presence of high NFκB activity in the cancer cells as a strategy for activating NFκB-dependent CRISPR/Cas13a in colon tumors to suppress oncogenes by therapeutic AAV (adeno-associated virus) vectors (43). PD-L1 expression in cancer cells and tumor associated myeloid cells promote tumor cell survival by preventing lymphocyte activation (44). PD-L1 is hence a major target for immunotherapy using immune-checkpoint inhibitors. Interestingly, PD-L1 expression is NFκB-dependent, is upregulated during EMT in lung cancer cells and over-expression of PD-L1 in tumors is often associated with the upregulation of EMT markers (45, 46).

TNFα/NFκB is an important SMAD-independent signaling pathway that modulates cancer cell EMT, driven by the cocktail of secreted factors released by cancer cells and tumor-associated macrophages (47). Unlike in TNFα-dependent cell death, we do not see the involvement of RIPK1 activation in 5-ITu-mediated suppression of TGFβ-induced EMT. While the sensitizing effect of 5-ITu on necroptosis is linked to the inactivation of IKK-dependent checkpoint on RIPK1 activation, the effect on EMT is independent of RIPK1 and potentially linked to NFκB-dependent transcription of EMT genes like *SNAI1*. This is supported by our observation that siRNA-mediated downregulation of IκBα abolishes the effect of 5-ITu on TGFβ-induced EMT marker expression. Interestingly, we identified a 4-gene signature defined by (*i*) TNF/NFκB-dependent expression, (*ii*) regulation by TGFβ signaling and (*iii*) sensitivity to 5-ITu, which is effective in predicting disease-free survival in lung and pancreatic cancer. A previous study reported a larger gene expression signature for prostate cancer prognosis, which consisted of 21 NFκB-dependent genes (47). Another gene signature of prognostic value identified for low-grade gliomas also consisted of 14 NFκB-dependent genes (48). We have focused on lung/pancreatic cancer, as our cell culture data is derived from cancer cells of pancreatic and lung cancer origin. However, this approach and the 4-gene signature identified in our study could be applicable to other forms of cancer.

5-ITu was discovered as an adenosine kinase inhibitor and was later established as a chemotherapeutic molecule with antineoplastic properties in mouse model *in vivo* (6, 7). While some effects of 5-ITu, including the blockade of SARS-CoV2 replication is associated with its ability to inhibit adenosine kinase activity, other effects are independent of adenosine kinase inhibition (8). Later studies established ERK2, Haspin and several others as protein kinase targets of 5-ITu (10, 13). The role of 5-ITu in TNFα and TGFβ signaling is indeed independent of adenosine kinase inhibition. Our earlier efforts to identify the direct kinase target of 5-ITu, relevant to its effect on TNF-induced cytotoxicity was unsuccessful, despite identifying potential 5-ITu binding targets, from a panel of 100 kinases (16). Using *in vitro* kinase assays, we have now identified IKK2/IKKβ as a direct protein kinase target of 5-ITu. NFκB reporter assays and quantitative kinase activity assays prove that 5-ITu is an efficient IKKβ inhibitor with an IC_50_ <300 nM *in vitro* and in cells, which is comparable to that of other IKK inhibitors including BMS345541. It should be noted that Haspin is the only protein kinase with a lower known IC_50_ value for 5-ITu *in vitro* (49). However, cellular inhibition of Haspin activity usually requires concentrations in the range of 300 to 1000 nM 5-ITu (50). These results indicate that the effect of 5-ITu on TNFα-induced RIPK1 activation and TGFβ-induced EMT are probably mediated by direct inhibition of the IKK1/2 activity. Incidentally, IKKs were not included in the earlier screens for 5-ITu targets (13, 16). While several other targets of 5-ITu, including protein kinases are relevant to chemotherapeutic intervention against cancer, the sensitization of cancer cells to TNF/smac-mimetics induced necroptosis and interference with EMT are interesting candidate features for anticancer drugs. Further *in vivo* studies probing the effect of 5-ITu alone and in combination with smac-mimetics will be necessary to delineate the efficacy and mechanistic contribution of RIPK1-dependent necroptosis and EMT inhibition on the anti-tumor activity of 5-ITu and/or analogous compounds.

## Experimental procedures

### Cell culture and treatments

PANC1 and HEK293T cells were cultured in DMEM High glucose media (#AL007A, HiMedia), and A549 cells were maintained in DMEM/F12 (#AL215A, HiMedia,) supplemented with 10% fetal calf serum (FCS) and 1x antibiotic-antimycotic solution (#15240062, Gibco) at 37 °C, in a humidified atmosphere supplemented with 5% CO_2_. Cells were treated as indicated in 10 cm dishes (cell-cycle analyses), 12-well plates (for immunoblot analyses, siRNA-mediated knockdown), six-well plates (RNA isolation), 24-well plates (reporter assays) or 96-well plates (viability, cytotoxicity and wound-healing assays). Cells were pretreated with inhibitors or DMSO as a solvent control before TNFα and/or TGFβ stimulation wherever indicated. All treatments were performed in normal growth medium for the respective cell lines.

### Antibodies and reagents

N-Cadherin/CDH2 (#13116), E-Cadherin/CDH1 (#3195), SNAIL/SNAI1 (#3879), ZEB1 (#3396), Vimentin (#5741), pS^536^-p65 (#3033), NF-κB p65 (#8242), IKKβ (#8943), SMAD2/3 (#3102), pS^465/467^-SMAD2/p S^423/425^-SMAD3 (#8828), IkBα (#4814), and pS^32/36^-IkBα (#9246) antibodies were from Cell Signaling Technology (CST). Further antibodies used were against GAPDH (#MAB932Hu23, Cloud-Clone Corp.), α Tubulin (#12G10, DSHB), GST (#sc-138, Santa Cruz Biotechnology). Goat anti-mouse IgG (H + L)-HRP (#115-035-003), and goat anti-rabbit IgG (H + L)-HRP (111-035-045) secondary antibodies were from Jackson Immuno Research Laboratories. Alexa-488 Goat anti-Rabbit IgG (H + L) antibody was from Elabscience (#E-AB-1055) and Phalloidin iFluor 647 Conjugate (#20555, Cayman chemicals). Other chemicals and reagents used were: DAPI (#80145, Sisco Research Laboratories), ATP (#RM439, HiMedia), DiR (DiIC18) (#60017, Biotium Inc.). Negative control siRNA and three independent siRNAs targeting *NFKBIA* were obtained from MedChemExpress (#HY-RS09260).

The source and working concentrations (unless stated otherwise in the figures or figure legends) used for the stimulants and inhibitors were: recombinant human TGFβ (#100-21-10, Peprotech, 5 ng/ml) and recombinant human TNFα (#PHC3016, Gibco, 10 ng/ml), 5-Iodotubercidin (#HY-15424, 5–10 μM), IKKα/β inhibitor BMS345541 Hydrochloride (#HY-10518, 5 μM) (IKKβ IC_50_ = 0.3 μM, IKKα IC_50_ = 4 μM) (25), SB431542 (#HY-10431, 10 μM), RIPK1 inhibitor Nec-1 (#HY-15760, 50 μM), GSK481 (HY-100131, 5 μM), and ABT-702 dihydrochloride (#HY-103161, 5–10 μM) were obtained from MedChemExpress.

### Analysis of cell viability and cell death

A549 and PANC1 cells were seeded in triplicates in 96 well plates in 100 μl media per well. The cell numbers were optimized as 6000 cells/well for both the cell lines for 48 h treatment. Next day, cells were treated with various indicated combinations of reagents. For measuring cell death by Sytox Green assay (#S7020, Invitrogen, 5 mM stock in DMSO), Sytox green solution was also added during the time of treatments at a final concentration of 0.25 μM and fluorescence measurements were made using a multimode reader (Cytation 5, BioTek). Cell viability was evaluated using WST-8 (#72465, SRL) reagent (51). A549 and PANC1 cells were seeded in triplicate in 96-well plates in 100 μl of media per well. Next day, cells were treated with various combinations of reagents as indicated for 48 h. WST-8 (10 μl/well) was added directly to the wells in complete medium and plates were then incubated in a cell culture incubator for 45 to 60 min at 37 °C and the absorbance was measured at 450 nm using a microplate reader (Cytation 5, BioTek).

### Quantitative scratch-wound healing assays

Fluorescence-based scratch wound healing assays were performed as described previously (22). In brief, DiR-labeled A549 or PANC1 cells were seeded in 96-well plates at a density of 40,000 cells per well. After 18 h of seeding, scratches were made using 10 μl tips. At the beginning of the experiment (t = 0) and after allowing migration for 18 or 24 h, images were acquired using the 800 nm channel (42 μm resolution) using a near-infrared fluorescence scanner (Odyssey DLx, LICORbio). Scans were converted to 8-bit images and analyzed using ImageJ, and data were represented as migration indices.

### Cell cycle analyses

A549 cells (9 × 10^5^ cells) were seeded in 10 cm dishes and were treated with TGF-β and/or 5-ITu (5 μM) as indicated for 24 h. The cells were harvested, washed with 1x phosphate-buffered saline (PBS), and fixed in ice-cold 90% ethanol. After fixation, cells were washed with 1x PBS, resuspended in the staining solution (100 μg/ml RNase A and 100 μg/ml Propidium Iodide in 1x PBS), and incubated in the dark for 30 min. DNA content was analyzed using a BD Accuri C6 Plus flow cytometer at a low flow rate, and cell cycle distribution was plotted and quantified using the Floreada.io software (https://floreada.io, accessed in August 2025).

### NFκB-luciferase reporter assays

HEK293T cells were transfected using polyethylenimine reagent (#408727, Sigma-Aldrich) with reporter plasmids followed by media change after 6 h. A 3x κB luciferase reporter construct (encoding firefly luciferase, a gift from Dr Mark Windheim, Hannover Medical School) (52) and pGL4.70-Renilla luciferase (normalisation control, Promega) were used. 18 hours post-transfection, cells were treated with TNFα or TGFβ along with 5-ITu (5 μM) and BMS345541 (5 μM) for additional six hours. For IC_50_ determination, experiments were performed with a range of 5-ITu concentrations (0.02, 0.1, 0.5, 1, 2.5 and 5 μM). The plates were lysed 24 hours post transfection in 1x Passive Lysis Buffer (#E1941, Promega). Reporter assays were performed using in house buffers as reported previously (53), and luminescence measured using a multi-mode reader (Cytation 5, BioTek). IC_50_ values were calculated using GraphPad Prism 11.0.0 (GraphPad Software Inc) using non-linear regression equation and setting the parameter to [inhibitor] *versus* response-variable slope (four parameters).

### *In vitro* kinase assays

For *In vitro* kinase assays, 20 ng recombinant GST IKKβ (#PV3836, Invitrogen) and 1 μg recombinant IκBα (#BB-SPP40, Bio Bharati LifeScience) were used in a reaction volume of 25 μl. The kinase was incubated with inhibitors at indicated concentrations (0.02, 0.1, 0.5, 1, 2.5 and 5 μM inhibitors for IC_50_ determination) at 30 °C for 5 min followed by addition of substrate and ATP mix, and was incubated for 30 min at 30 °C. The final reaction buffer consisted of 20 mM HEPES, 20 mM β-glycerophosphate, 10 mM NaF, 10 mM MgCl_2_ and 25 μM ATP. The reaction was stopped by adding 5X SDS loading dye and monitored by immunoblotting. Immunoblots were quantified, and IC_50_ values were calculated as described earlier using GraphPad Prism 11.0.0.

### siRNA-mediated knockdown of IκBα

A549 cells were seeded in 12-well plates at a density of 1 × 10^5^ cells per well and were transfected with 25 nM siRNAs using RNAiMAX transfection reagent (#13778030, Invitrogen), following the manufacturer's protocol. Three independent siRNAs targeting human IκBα gene (*NFKBIA*) and a negative control siRNA were used. Media was changed to complete DMEM/F12 after 8 h of transfection. Forty hours post-transfection, cells were treated with TGFβ and 5-ITu (5 μM) or a solvent control (DMSO) for 8 h. Cells were analyzed by immunoblotting to verify knockdown and quantitative real-time PCR to monitor EMT marker expression.

### RNA isolation, cDNA synthesis, and qPCR analyses

A549 and PANC1 cells were treated for 24 h in 6-well plates, total RNA was isolated using the NucleoSpin RNA extraction kit (Macherey & Nagel), and cDNA was synthesized using a cDNA synthesis kit (BB-E0043, Bio Bharati LifeScience,) using random hexamer primers according to the manufacturer's instructions. Real-time PCR quantitation was performed using TB Green Premix Ex Taq II (Tli RNase H Plus, Takara) on a CFX96 system (Bio-Rad). Gene expression was normalized to *GAPDH* or Cyclophilin/*PPIA*. The primer sequences used are listed in the Table S1.

### Western blotting

After 24 h treatments with TGFβ and/or inhibitors, cells were lysed in kinase-assay lysis buffer (20 mM Tris, 1 mM Na_3_VO_4_, 1 mM EDTA, 1 mM EGTA, 10 mM β-glycerophosphate, 50 mM NaF, 1% Triton X-100) supplemented with protease inhibitor cocktail (#11873580001, Sigma-Aldrich, and phosphatase inhibitor cocktails (#04906845001, Phosstop, Roche), and total protein quantified by Bradford assay (#5000205, Bio-Rad) before processing for immunoblotting. SDS-PAGE (10%) gels were used for protein separation followed by western blotting using nitrocellulose membrane (#10600001, Bio-Rad). Membranes were blocked with 5% powdered skim milk in PBS with 0.1% Tween 20 for 1 h at room temperature (RT), followed by incubation with the primary antibodies at 4 °C overnight. Blots were washed, incubated with secondary antibodies for one hour at RT and were developed using ECL reagents (Westarnova 2.0, Cyanagen; Clarity Max, Bio-Rad); bands were visualized using a Fusion Solo 6S chemiluminescence imager (Vilber). Densitometric analysis was performed using ImageJ v. 1.54 (https://imagej.nih.gov/ij/) and normalized against respective loading controls. For quantification of phospho-signals, bands were normalized to the total protein signals.

### Immunostaining and microscopy

For obtaining phase-contrast images, A549 and PANC1 cells were seeded in 12-well plates and treated with TGFβ only and co-treated with 5-ITu and SB42 or with 5-ITu only for 24 h. Live phase-contrast images were captured using a camera attached to the microscope (#PZQ-106S, Quasmo, India). For Immuno-fluorescence staining, cells were seeded on poly-L-lysine precoated glass coverslips and processed as reported previously (54). Primary antibodies against CDH1 were used at a 1:200 dilution in 1% BSA–PBS for 2 h at RT. Alexa-488-labeled secondary antibodies and fluorescent labeled Phalloidin were used at 1:250 dilution in 1% BSA-PBS at RT for 45 min, followed by DAPI (500 ng/ml in PBS) staining for 10 min at RT. Imaging was performed using a Leica TCS SP8 confocal microscope with standard settings.

### *In silico* data analyses

HALLMARK_TGF_BETA_SIGNALING and HALLMARK_TNFA_SIGNALING_VIA_NFKB genesets were obtained from the Molecular Signature Database (www.gsea-msigdb.org) (31, 32). Venn diagrams were created using Venny 2.1 (https://bioinfogp.cnb.csic.es/tools/venny/) (55). We used the GEPIA2 server (http://gepia2.cancer-pku.cn/) (56) to access TCGA (https://www.cancer.gov/tcga) data and perform survival analyses (overall survival and disease-free survival) of TCGA lung cancer (lung adenocarcinoma and squamous cell carcinoma) and pancreatic cancer data. Patient samples were categorized into high-expression and low-expression groups based on the median expression cutoffs. Survival curves were plotted using the Kaplan-Meier method, and differences were evaluated using the log-rank test. The Hazard Ratio (HR) and 95% confidence intervals (CI) were calculated with the Cox proportional hazards model, using the standard parameters of the GEPIA2 server. To check whether EMT marker genes (*CDH1*, *CDH2*, *SNAI1*, *SNAI2*, *TWIST1*, *TWIST2*, *VIM*, *ZEB1 and ZEB2*) are direct targets of NFκB-dependent transcription, Gene Transcription Regulation Database (https://gtrd.biouml.org) was used to detect ChIP-Seq-based binding sites for RELA/p65, NFKB1/p50 and REL/c-Rel in the −1000 to +100 promoter regions of these genes. *NFKBIA* promoter was used as a positive control target of NFκB signaling.

### Statistics and reproducibility

All cell death and viability assays were performed in biological triplicate samples, and the data presented are representative of three independent experiments. All Immunoblot results presented in figures are representative results from at least three independent experiments, unless indicated otherwise in the figure legends. Migration assays were performed with a minimum of four biological replicates and the data presented are representative of at least two independent experiments. The calculations, statistical analyses and graphs were performed using Microsoft Excel. Two-tailed unpaired *t* test was used to calculate statistical significance for quantitative assays. The statistics and source data are presented in Table S2.

## Data availability

All data generated during this study leading to the findings presented here are included in this published article and its supplementary data files. The patient survival data from The Cancer Genome Atlas (TCGA) was accessed, and survival plots were generated directly using the GEPIA portal.

## Supporting information

This article contains supporting information.

## Conflict of interest

The authors declare that they have no conflicts of interest with the contents of this article.
