# Supplementary material for: 5-Iodotubercidin inhibits epithelial to mesenchymal transition by inhibiting IKK/NFκB-dependent gene expression
Source: J Biol Chem. 2026 Sep 12;302(11):113548. doi: 10.1016/j.jbc.2026.113548 (PMC13677950; doi:10.1016/j.jbc.2026.113548)

Supplementary Figure S1

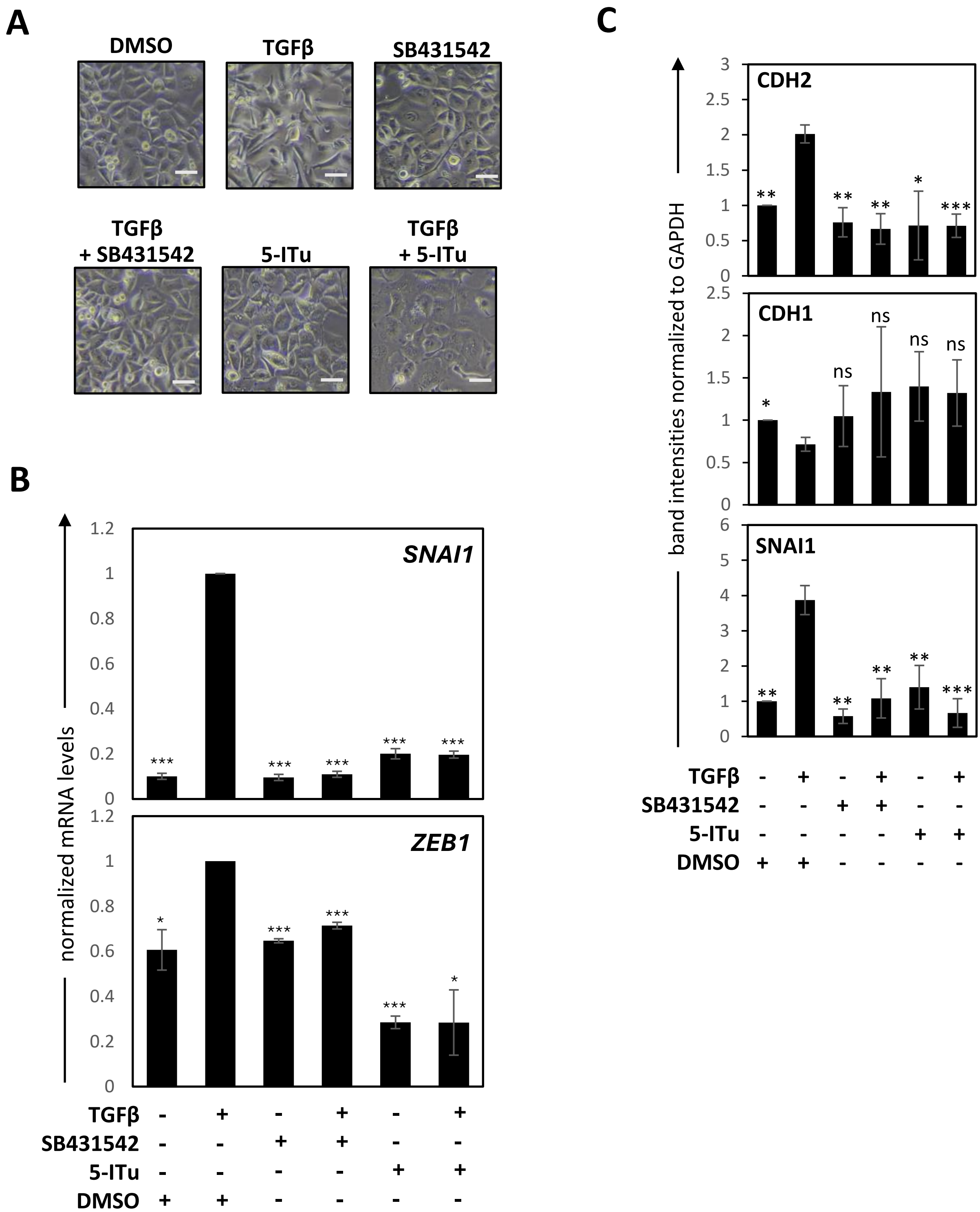

Supplementary Figure S2

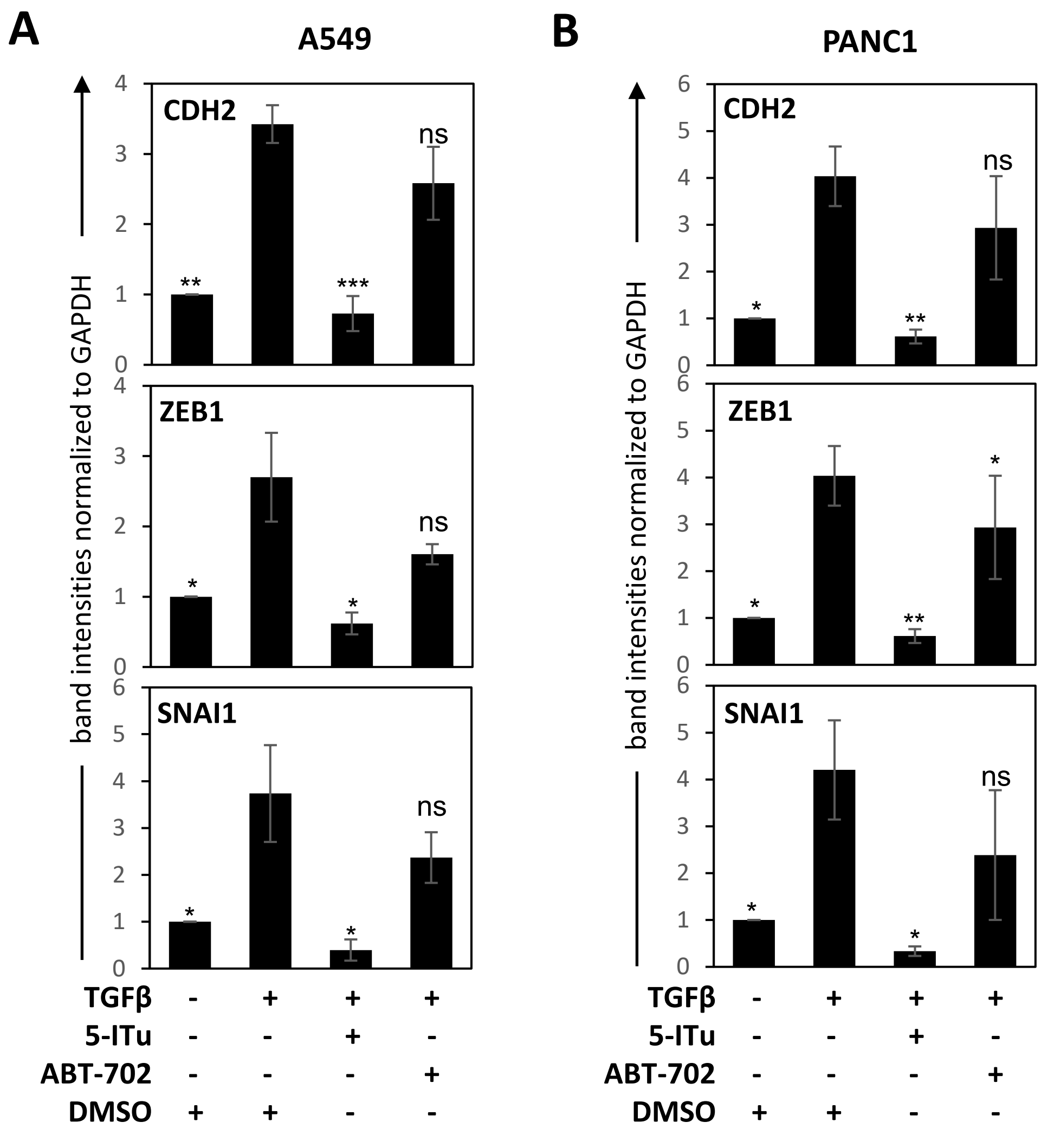

Supplementary Figure S3

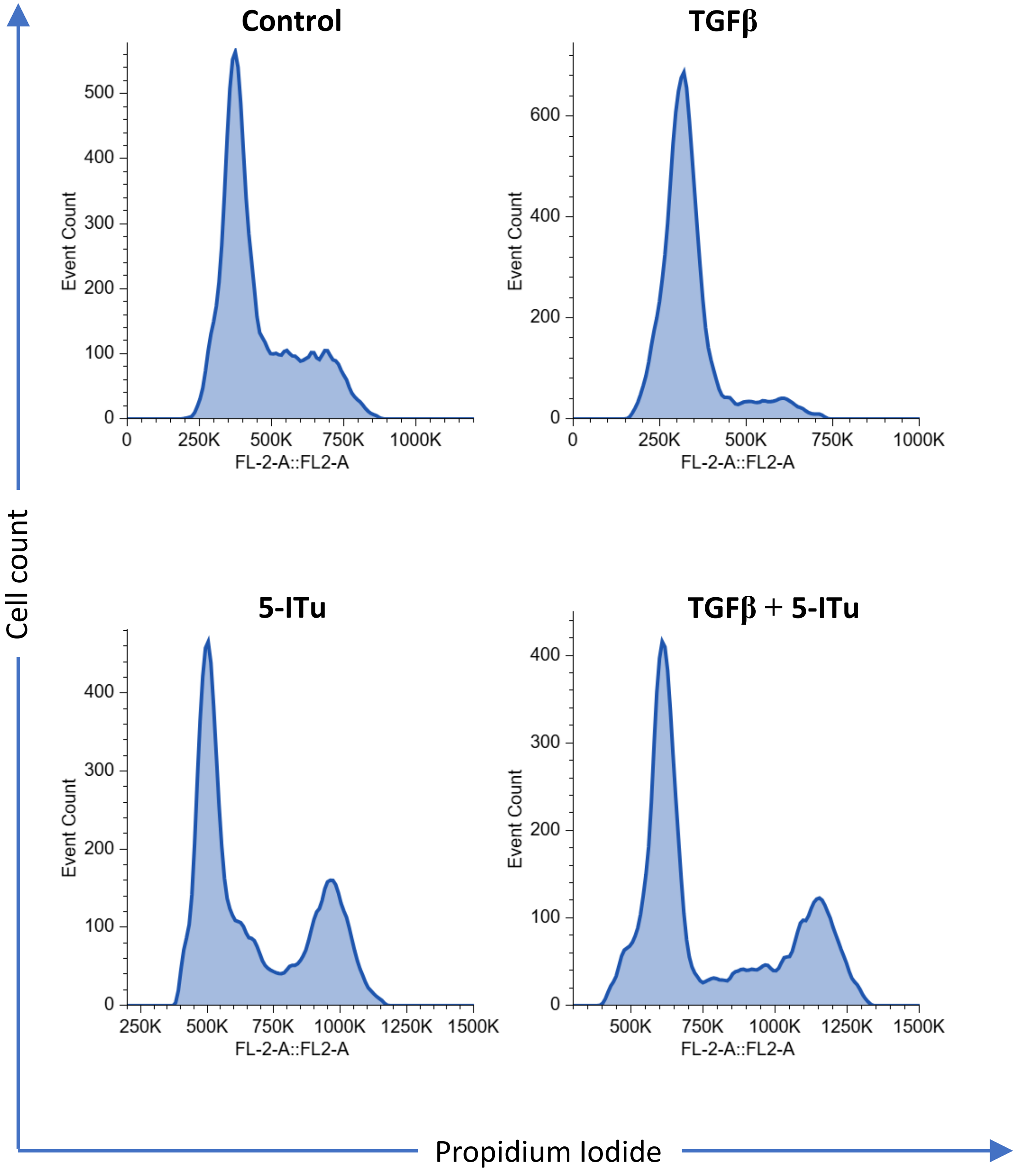

Supplementary Figure S4

A

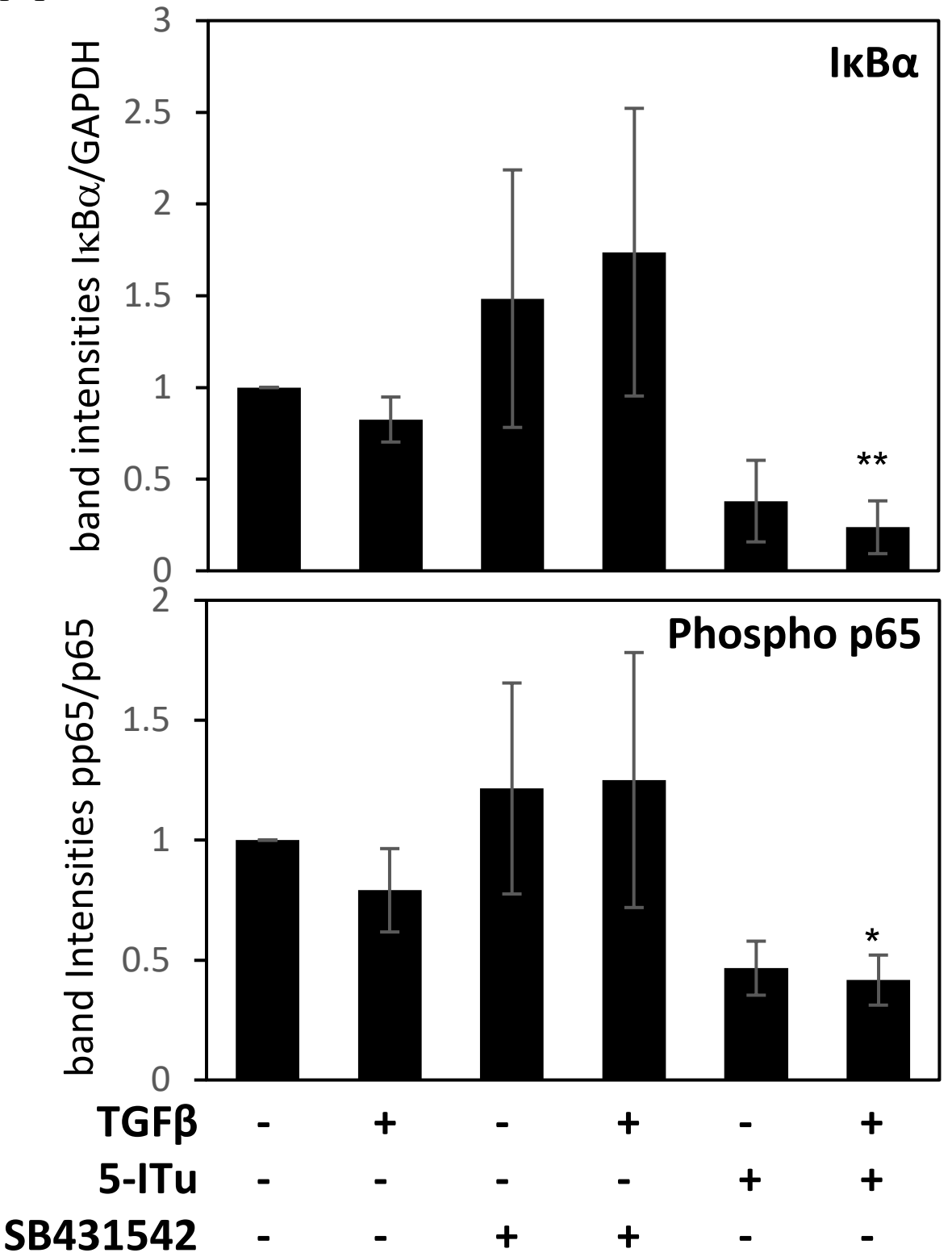

B

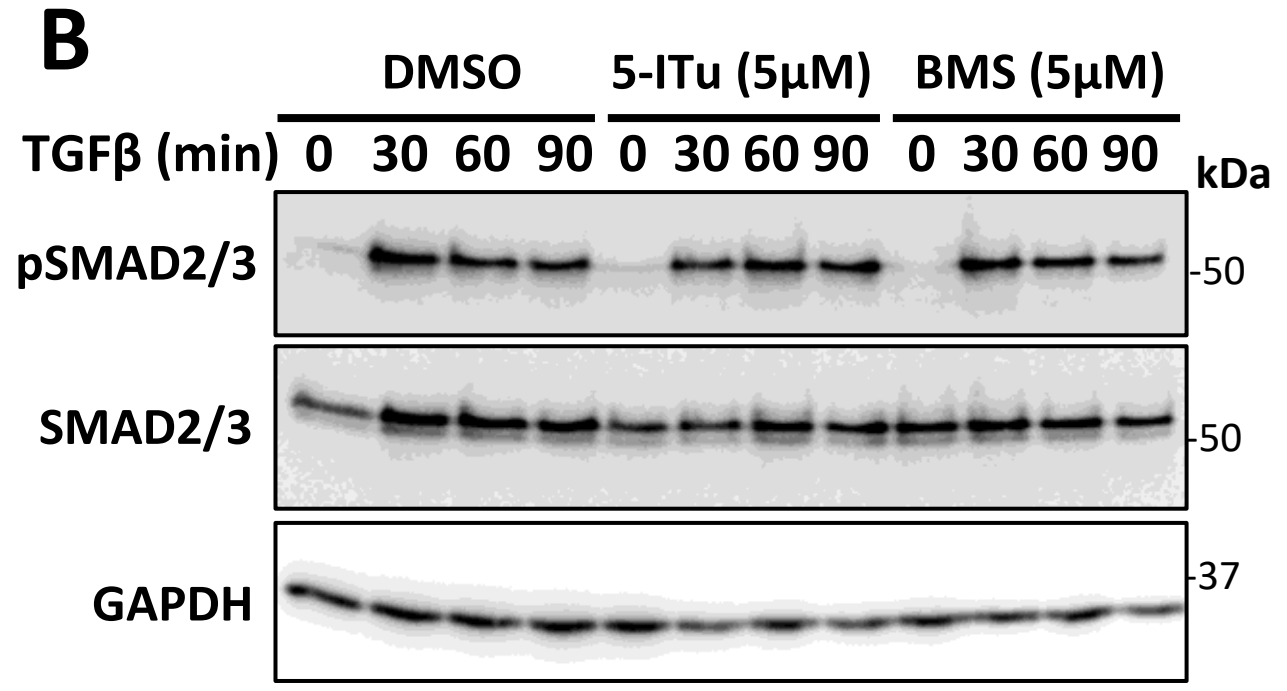

C

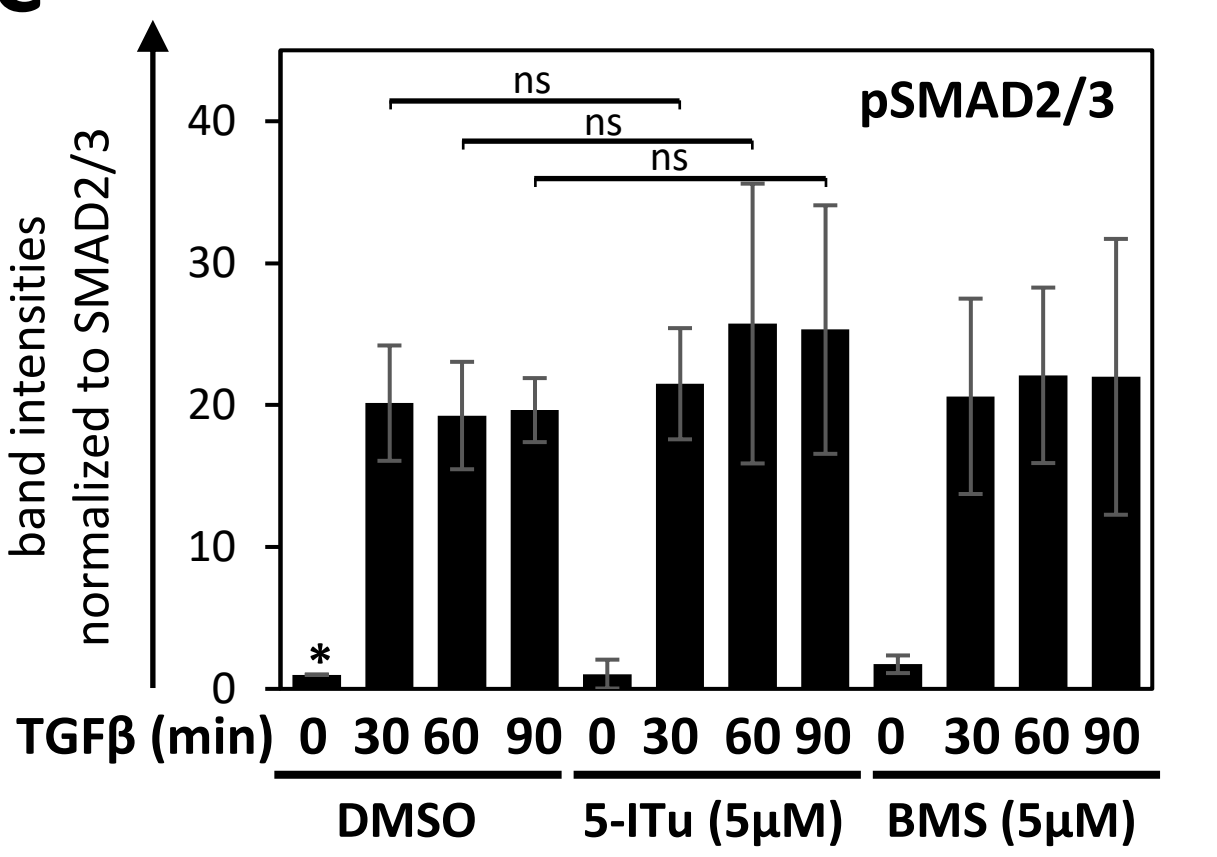

D

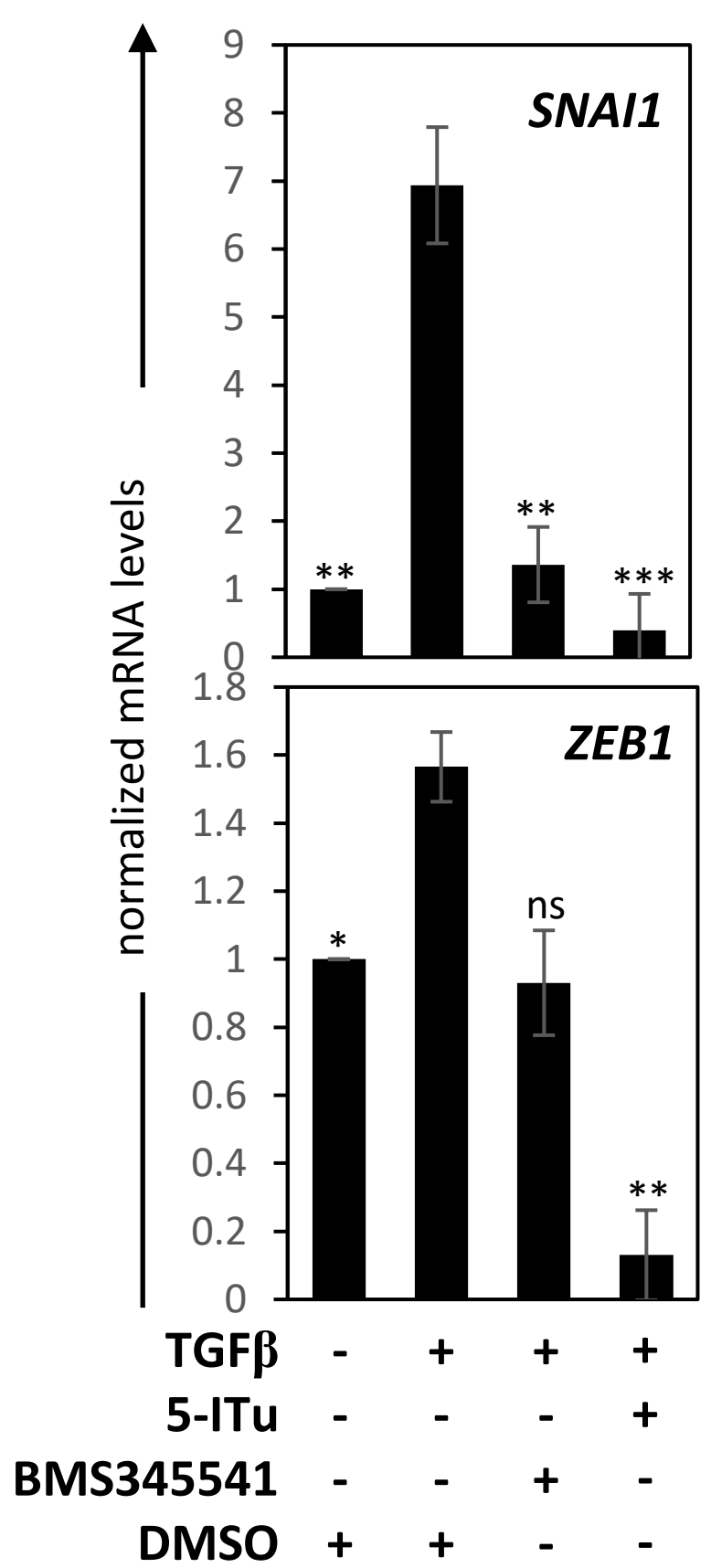

E

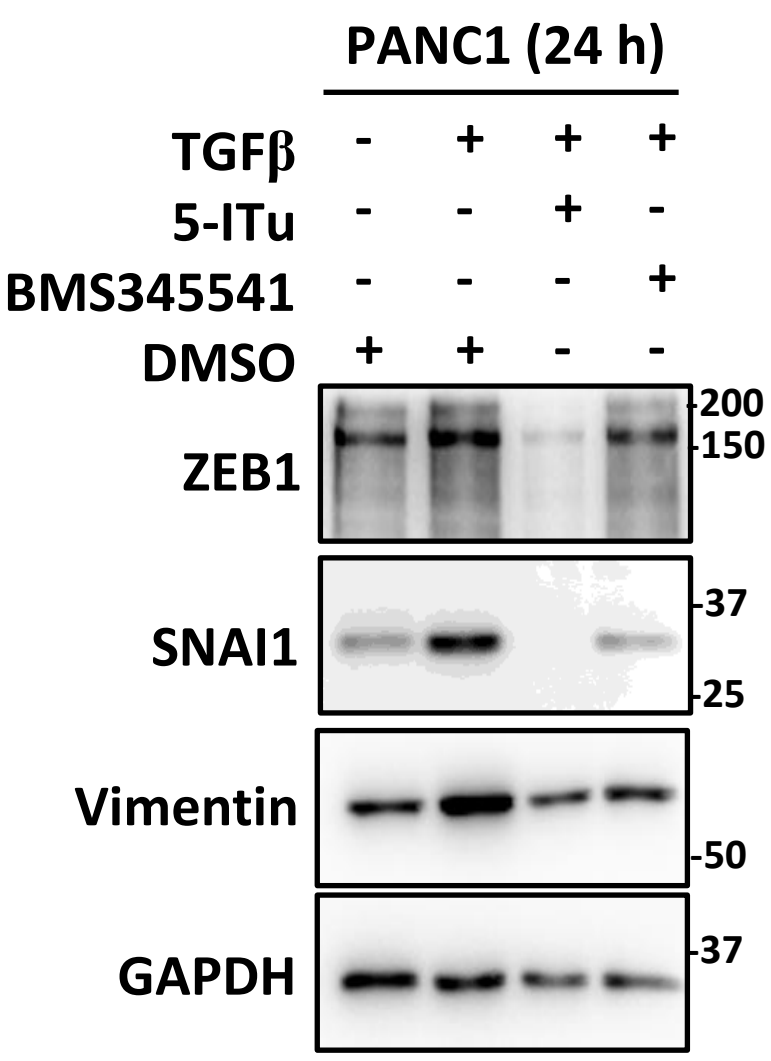

F

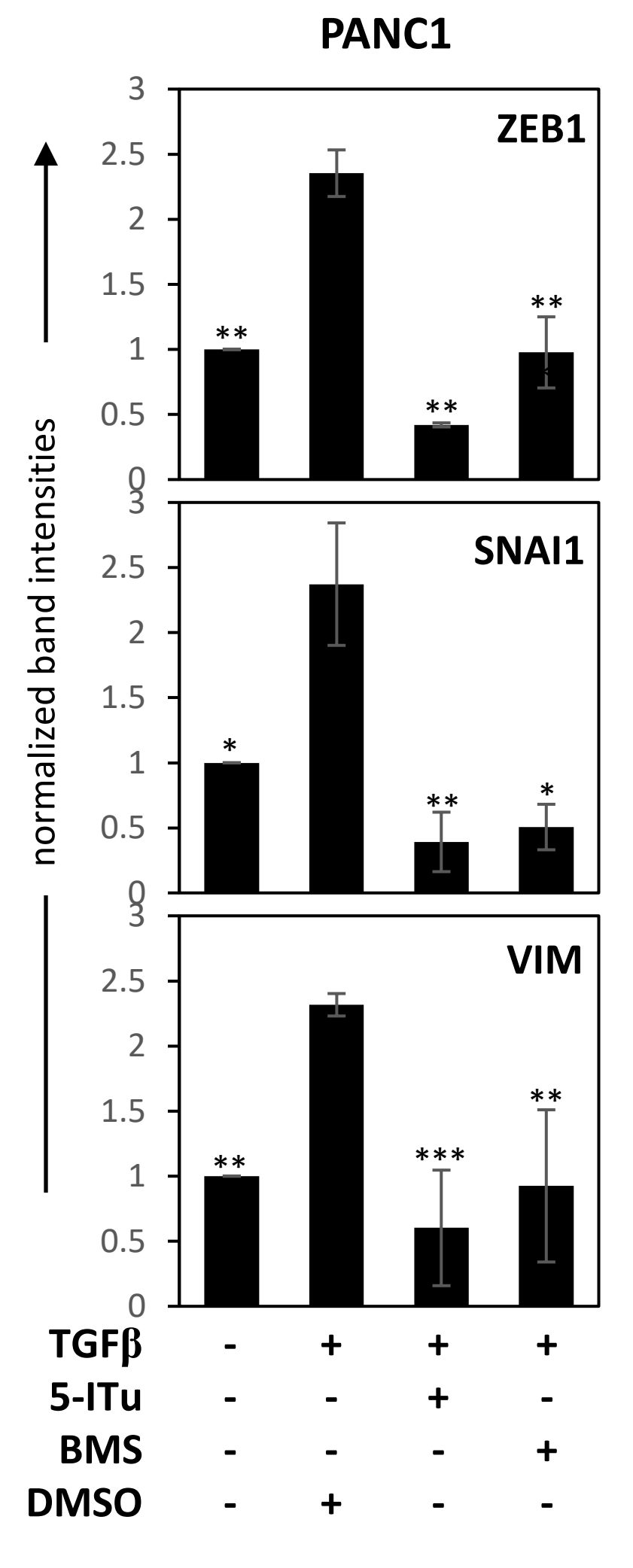

G

Gene symbol	Number of TF binding sites		
	RELA/p65	NFKB1/p50	REL/c-Rel
NFKBIA	7	3	2
VIM	9	1	3
ZEB1	8	2	2
SNAI1	6	0	1
ZEB2	4	2	1
SNAI2	4	1	0
TWIST1	4	0	0
CDH1	2	1	0
CDH2	2	0	0
TWIST2	1	0	0

Supplementary Figure S5

A

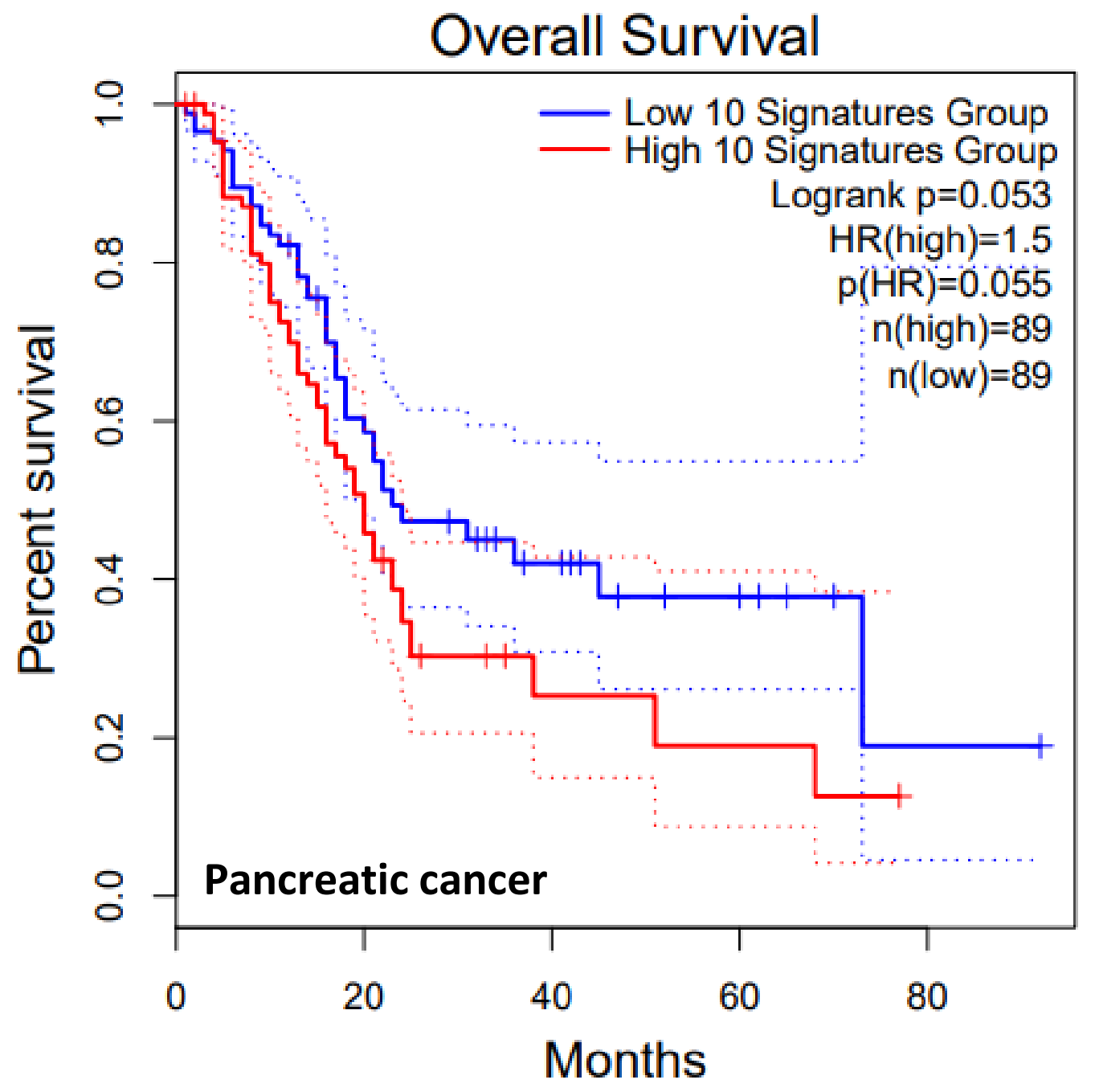

B

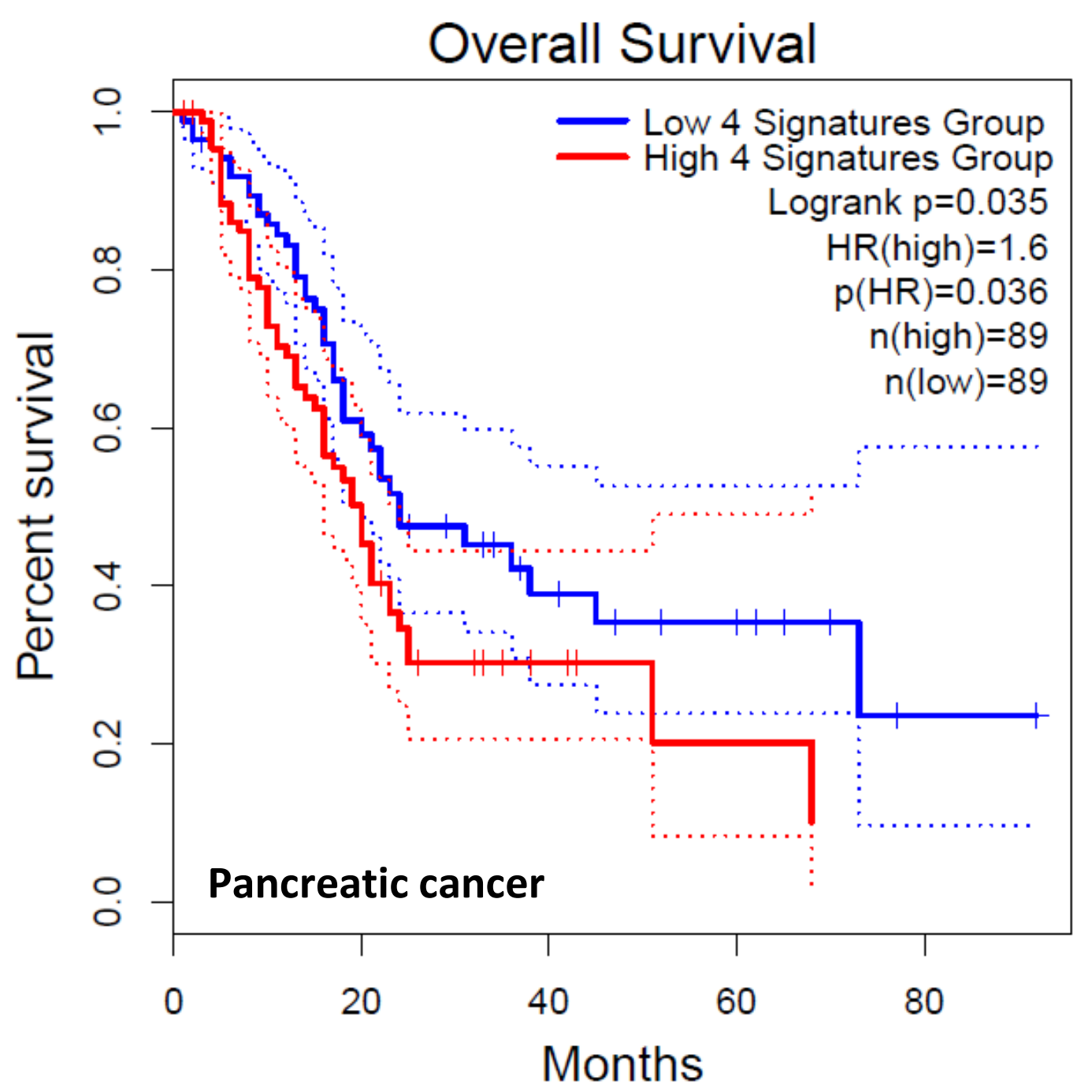

Supplement: Supplementary file 1 [file mmc2.pdf]
