# Supplementary material for: 5-Iodotubercidin inhibits epithelial to mesenchymal transition by inhibiting IKK/NFκB-dependent gene expression
Source: J Biol Chem. 2026 Sep 12;302(11):113548. doi: 10.1016/j.jbc.2026.113548 (PMC13677950; doi:10.1016/j.jbc.2026.113548)
Supplement: Supplementary file 2 [file mmc3.pdf]

Supplementary Information Legends

1. Supplementary Figures S1-S5 (single .pdf file)

Supplementary Figure S1. 5-ITu inhibits TGFβ-induced EMT in PANC1 cells. A. PANC1 cells were treated as indicated for 24 h and images were acquired using a phase-contrast 10x objective. Scale bars indicate 50 µm. **B.** PANC1 cells were stimulated with TGFβ in combination with indicated inhibitors or solvent control (DMSO) and EMT marker (*ZEB1, SNAI1*) expression was monitored by qPCR. **C.** Densitometric Analyses of the immunoblots shown in Figure 1D normalised against the indicated loading control (n=3, mean±SD) (* denotes p-value ≤ 0.05 and *** denotes p≤ 0.001).

Supplementary Figure S2. Adenosine kinase inhibition does not alter EMT marker protein levels. Immunoblots in Figure 2D and 2E, respectively, for A549 (A) and PANC1 (B) cells, treated with TGFβ along with 5-ITu or ABT702 were quantified and normalized to GAPDH control. (n=3, mean±SD) (* denotes p-value ≤ 0.05 and *** denotes p≤ 0.001).

Supplementary Figure S3. Cell-cycle analyses of 5-ITu-treated A549 cells. Representative flow-cytometry histograms for the data presented in Figure 3D. Cells were treated for 24h as follows: untreated control cells, cells treated with 5 µM 5-ITu, 5 ng/mL TGF-β or both, and fixed and stained with propidium iodide. DNA content was analysed by flow cytometry.

Supplementary Figure S4. 5-ITu inhibits NFκB signaling and not SMAD2/3 signaling to suppress Epithelial-Mesenchymal Transition. A. The graphs show the immunoblot quantification of the bands shown in Figure 4A. p^{S536}-p65 levels are normalized to total p65 signals, and IκBα bands are normalized to GAPDH control, respectively. **B & C.** A549 cells were treated with TGFβ for the indicated time points in the presence of solvent control (DMSO), 5-ITu (5 µM), or BMS (5 µM) and analyzed for phospho-SMAD2/3, SMAD2/3, and GAPDH control levels by immunoblotting (B). The bands are quantified, normalized and represented (C). **D-F.** PANC1 cells were treated as indicated and EMT markers were studied by qPCR (D) and Immunoblotting (E) followed by subsequent densitometric analyses (F**)** (* denotes p-value ≤ 0.05, ** denotes p-value ≤ 0.01 and *** denotes p-value ≤ 0.001). **G.** Number of binding sites for NFκB family transcription factors (TF) identified from ChIP-Seq datasets in the promoter regions of EMT marker genes are shown. *NFKBIA* gene is shown as a positive control.

Supplementary Figure S5. NFκB/5-ITu-dependent prognostic gene expression signatures in pancreatic cancer. A & B. TCGA pancreatic datasets were used to plot overall survival of pancreatic cancer patients based on the 10-gene signature mentioned in Figure 6A-B (A) or the NFκB-dependent, 5-ITu-sensitive 4-gene signature (B). Median expression values used to determine high/low expression in all survival plots and other statistical parameters are shown in the plots. The plots were generated using the GEPIA2 webserver.

2. **Supplementary Table S1. Sequence of primers used in the study.** Sequence details of qPCR primers used in the study (MS Excel file).

3. **Supplementary Table S2. Statistics and source data for the quantitative results.** The actual values (source data) and the *p*-values calculated for the quantitative data presented in the manuscript (MS Excel file)

4. **Supplementary Data File 2: Uncropped blots** (.pdf document)
