# Supplementary material for: 5-Iodotubercidin inhibits epithelial to mesenchymal transition by inhibiting IKK/NFκB-dependent gene expression
Source: J Biol Chem. 2026 Sep 12;302(11):113548. doi: 10.1016/j.jbc.2026.113548 (PMC13677950; doi:10.1016/j.jbc.2026.113548)
Supplement: Supplementary file 3 [file mmc4.pdf]

Supplementary Table S1: Oligonucleotide sequences used for real time qPCR			
Sl.No	Names	species	Primer Sequences
1	hSMAD3-Fwd	Human	TCGAGCCCCAGAGCAATATT
2	hSMAD3-Rev	Human	CGTCCATGCTGTGGTTCATC
3	SERPINE1-Fwd	Human	TCGAGGTGAACGAGAGTGGCA
4	SERPINE1-Rev	Human	AAGGACTGTTCCTGTGGGGTTGT
5	hTGIF1-Fwd	Human	GACATTCCCTTGGACCTT TCT
6	Htgif1-Rev	Human	TACAGCCAATCCCGAAGAATC
7	Hpmepa1-Fwd	Human	TGTCAGGCAACGGAATCCC
8	hPMEPA1-Rev	Human	CAGGTACGGATAGGTGGGC
9	hID2-Fwd	Human	ATGAAAGCCTTCAGTCCGGTG
10	hID2-Rev	Human	AGCAGACTCATCGGGTCGT
11	hPPP1R15A-Fwd	Human	GAATCAAGCCACGGAGGATA
12	hPPP1R15A-Rev	Human	CAGGGAGGACACTCAGCTTC
13	hJUNB-Fwd	Human	CGACCACCATCAGCTACCTC
14	Hjunb-Rev	Human	GTCTGCGGTTCCTCCTTGAA
15	hXIAP-Fwd	Human	TGGTATCCAGGGTGCAAATATCT
16	hXIAP-Rev	Human	GTAGTTCTTACCAGACACTCCTCA
17	hKLF10_fwd	Human	GCCAACCATGCTCAACTTCG
18	hKLF10_rev	Human	TGCAGTTTTGTTCCAGGAATACAT
19	hBMP2_fwd	Human	ACCCGCTGTCTTCTAGTGTTG
20	hBMP2_rev	Human	TTCTTCGTGATGGAAGCTGAG
21	hIFNGR2 Fwd	Human	GCAAGATTCGCCTGTACAACG
22	hIFNGR2 Rev	Human	GTCACCTCAATCTTTTCTGGAG
23	huCDH2_qPCR_F	Human	TGAGGAGTCAGTGAAGGAGTCA
24	huCDH2_qPCR_R	Human	CCAGTCTCTCTTCTGCCTTTGT
25	huCDH1_qPCR_F	Human	CCCGGGACAACGTTTATTAC
26	huCDH1_qPCR_R	Human	GCTGGCTCAAGTCAAAGTCC
27	sy-huSNAI1/122s	Human	TCGGAAGCCTAACTACAGCG
28	sy-huSNAI1/277as	Human	GCCAGGACAGAGTCCCAGA
29	huCyclophilin_qPCR_F	Human	GTCAACCCCACCGTGTTCTT
30	huCyclophilin_qPCR_R	Human	CTGCTGTCTTTGGGACCTTGT
31	huVIM_qPCR_F	Human	TGAGTACCGGAGACAGGTGCAG
32	huVIM_qPCR_R	Human	TAGCAGCTTCAACGGCAAAGTTC
33	sy-huZEB-1/s	Human	TTTCCCATTCTGGCTCCTAT
34	sy-huZEB-1/as	Human	GATGCTGAAAGAGACGGTGA
35	hu GAPDH_Fwd	Human	TGCACCACCAACTGCTTAGC
36	hu GAPDH_Rev	Human	GGCATGGACTGTGGTCATGAG
