# Supplementary material for: 5-Iodotubercidin inhibits epithelial to mesenchymal transition by inhibiting IKK/NFκB-dependent gene expression
Source: J Biol Chem. 2026 Sep 12;302(11):113548. doi: 10.1016/j.jbc.2026.113548 (PMC13677950; doi:10.1016/j.jbc.2026.113548)

Figure 1D

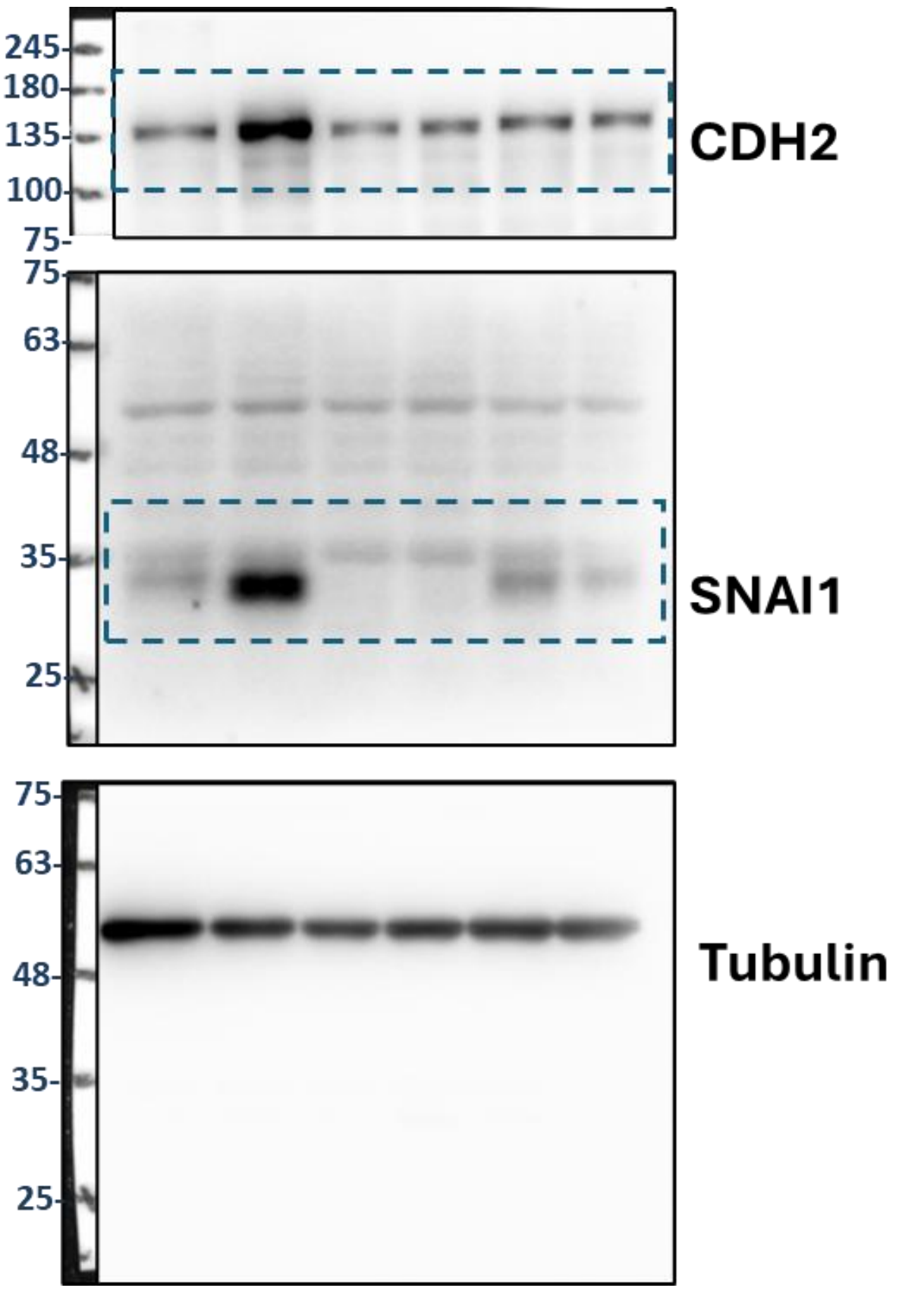
245
180
135
100
75
CDH2
75
63
48
35
25
SNAI1
75
63
48
35
25
Tubulin

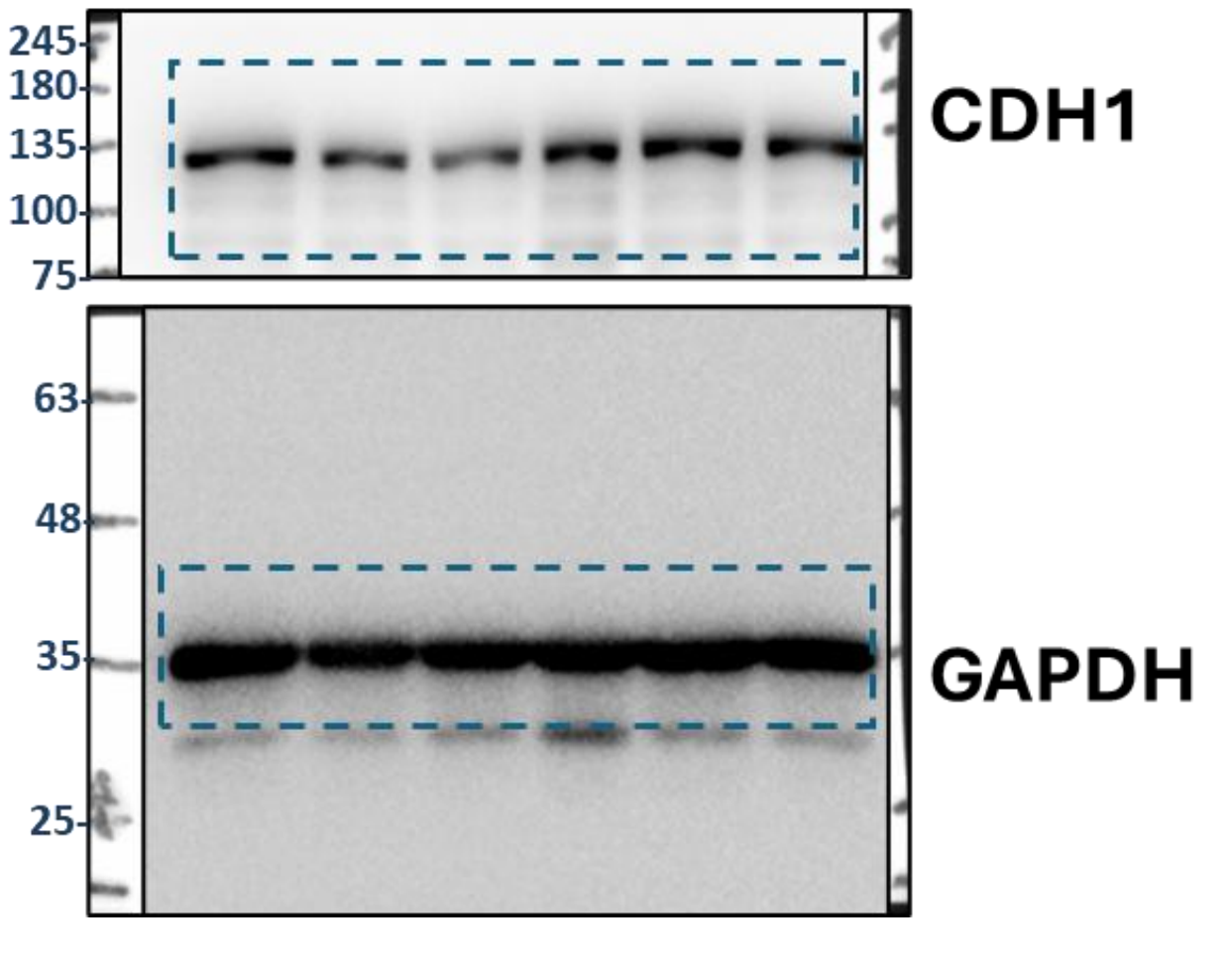
245
180
135
100
75
CDH1
63
48
35
25
GAPDH

Figure 2D

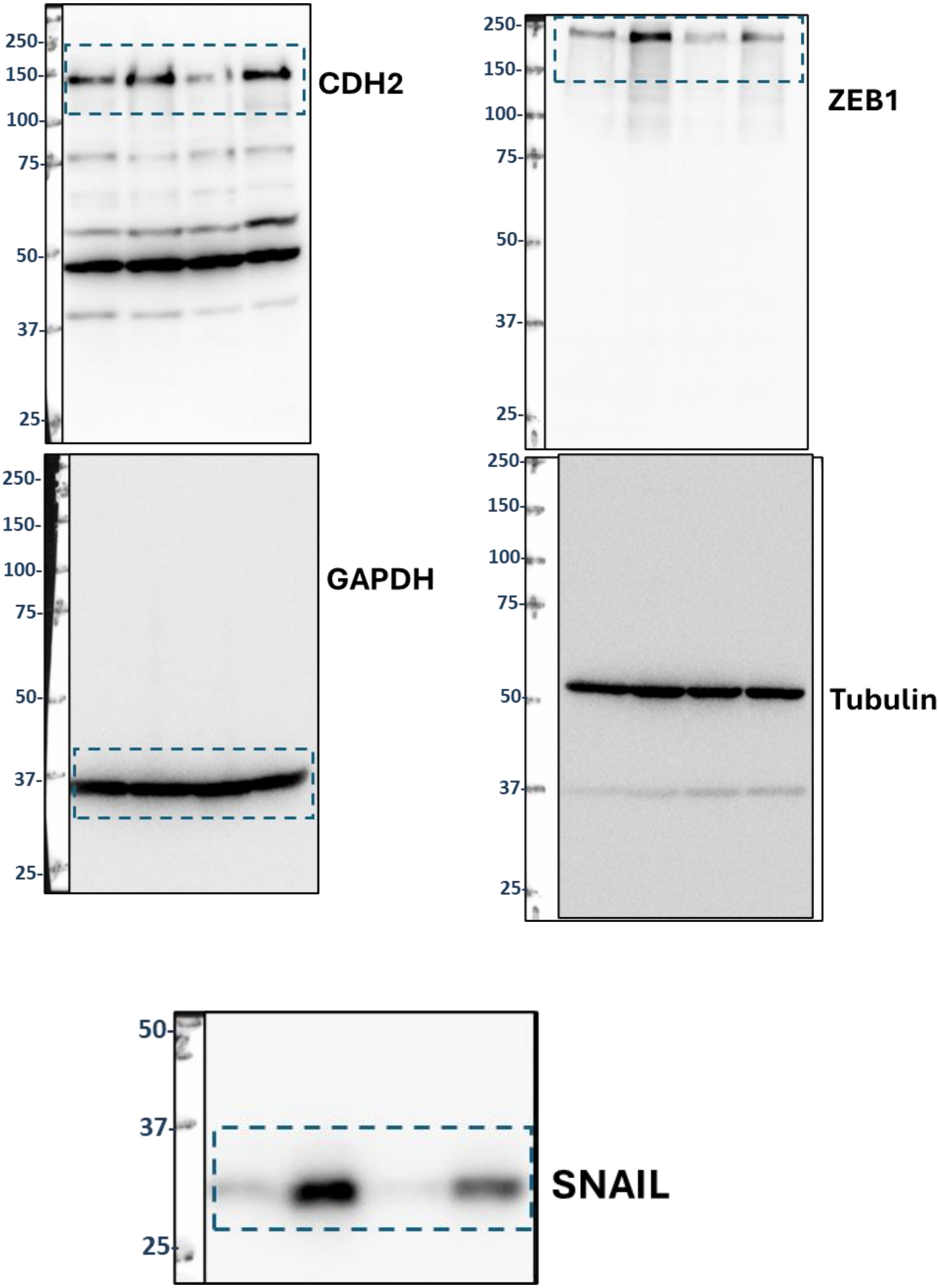

Figure 2E

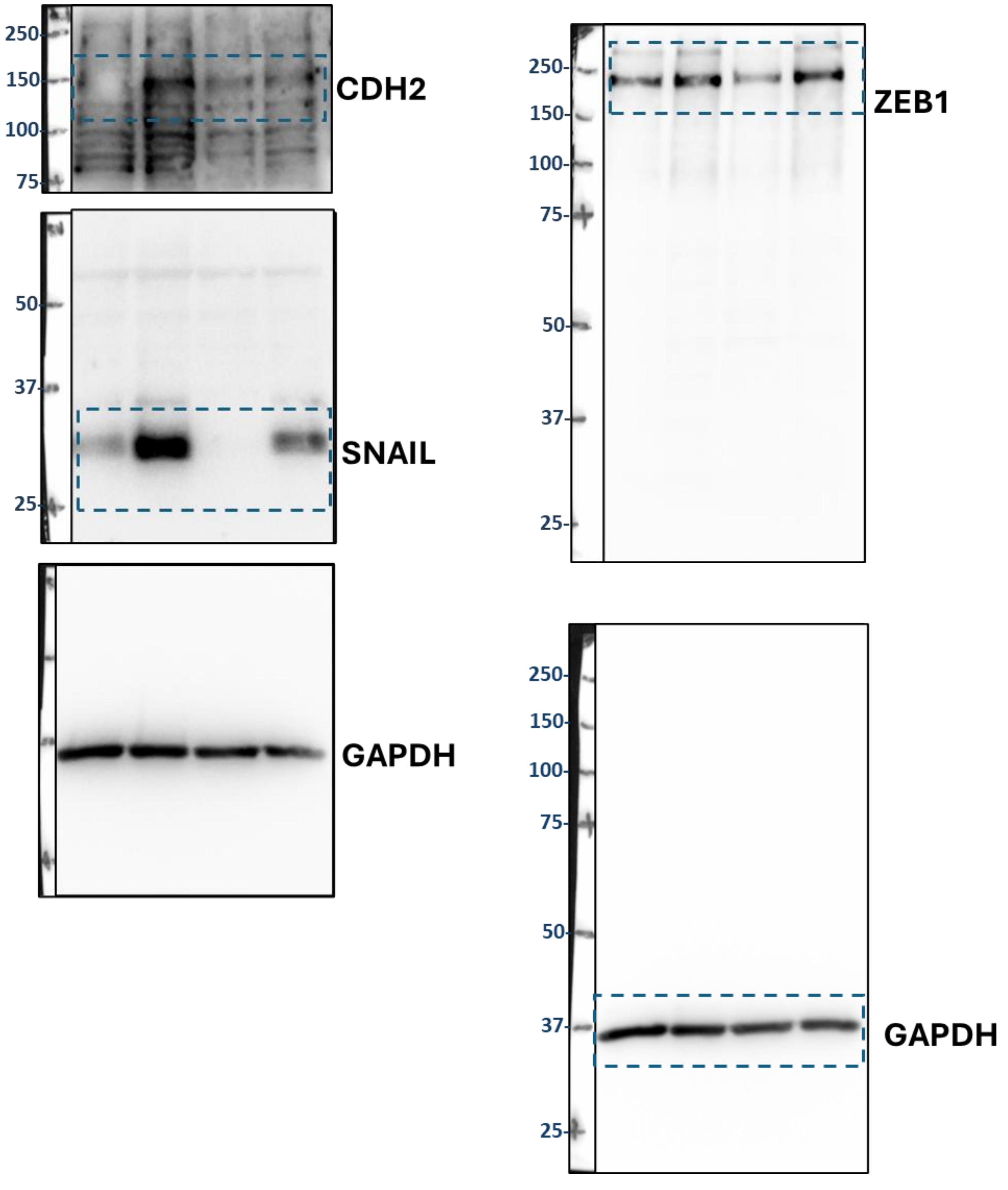
250
150
100
75
CDH2
50
37
25
SNAIL
GAPDH
250
150
100
75
50
37
25
ZEB1
250
150
100
75
50
37
25
GAPDH

Figure 4A

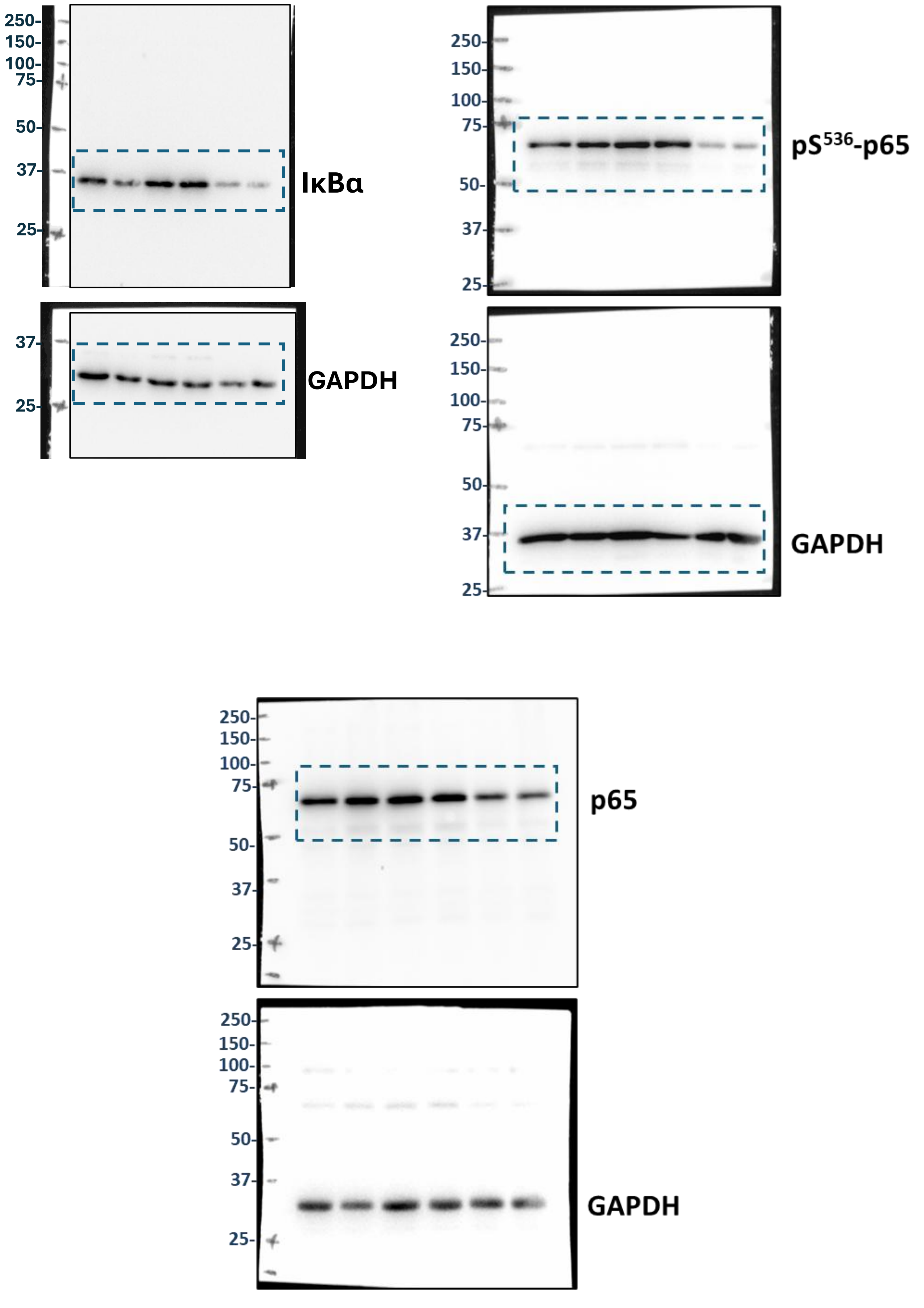
250-
150-
100-
75-
50-
37-
25-
IκBα
37-
25-
GAPDH
250-
150-
100-
75-
50-
37-
25-
pS536-p65
250-
150-
100-
75-
50-
37-
25-
GAPDH
250-
150-
100-
75-
50-
37-
25-
p65
250-
150-
100-
75-
50-
37-
25-
GAPDH

Figure 4D

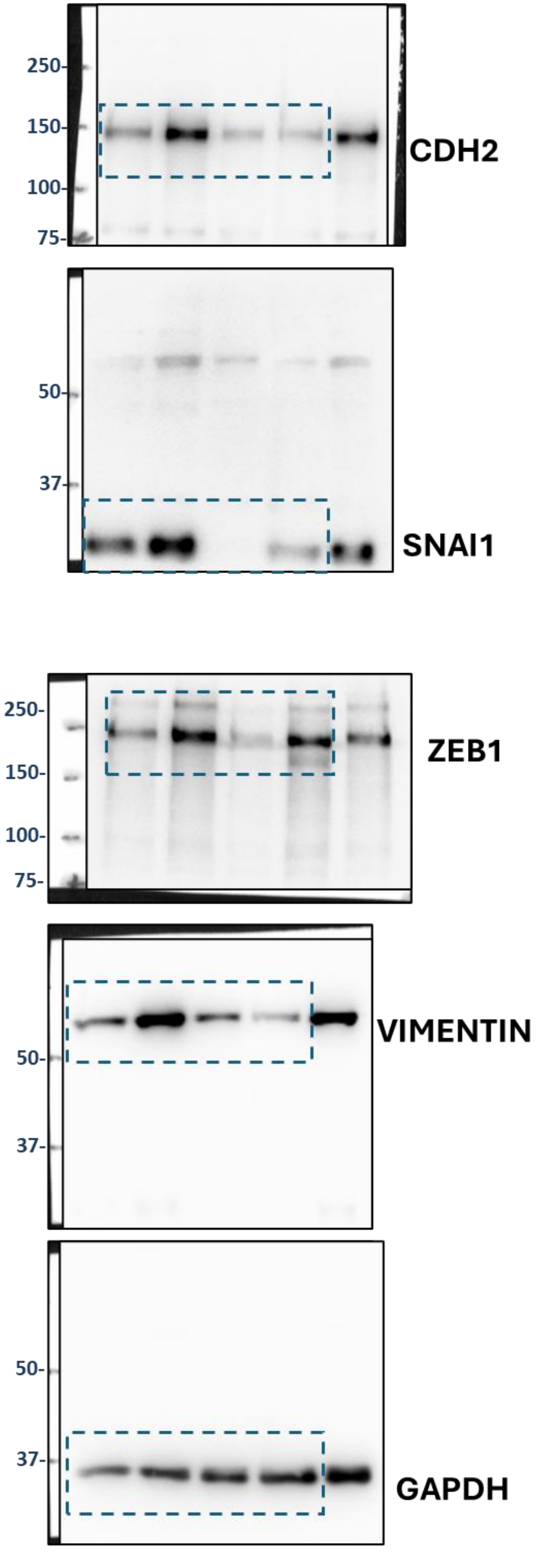
250-
150-
100-
75-
CDH2
50-
37-
SNAI1
250-
150-
100-
75-
ZEB1
50-
37-
VIMENTIN
50-
37-
GAPDH

Figure 5C

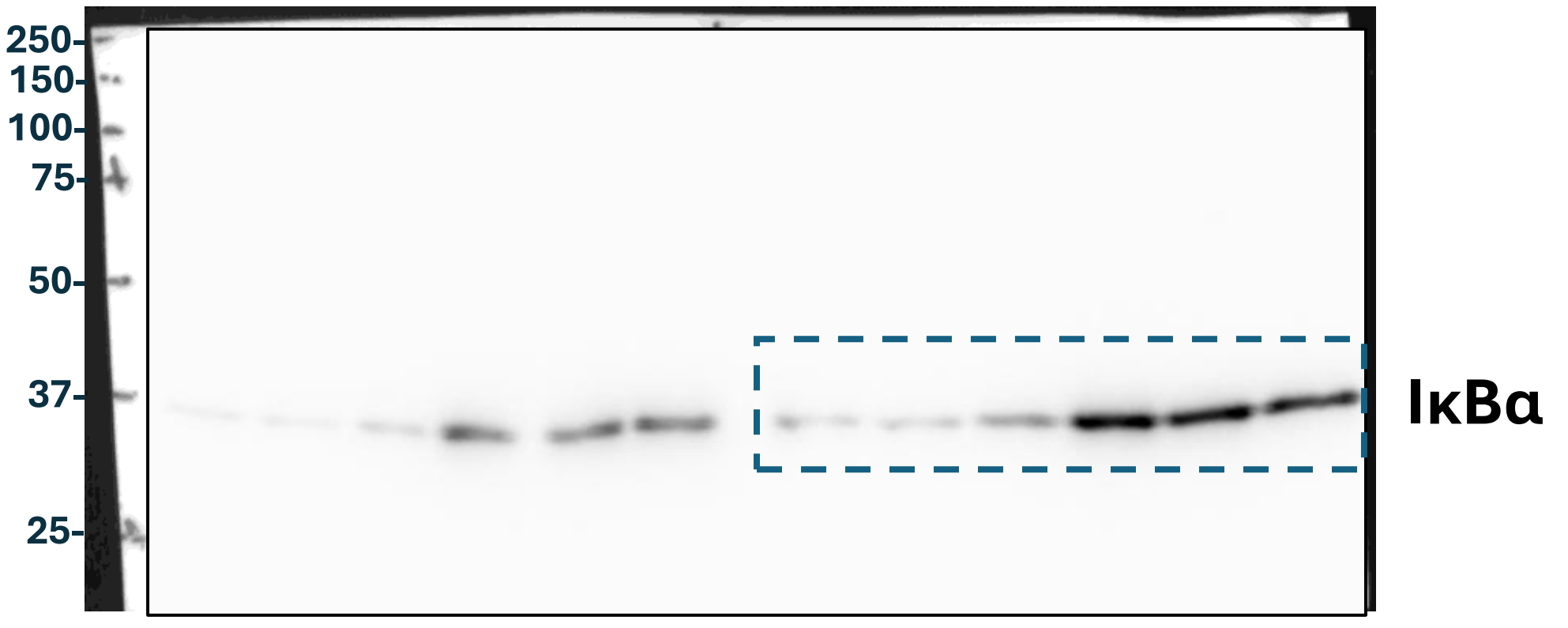

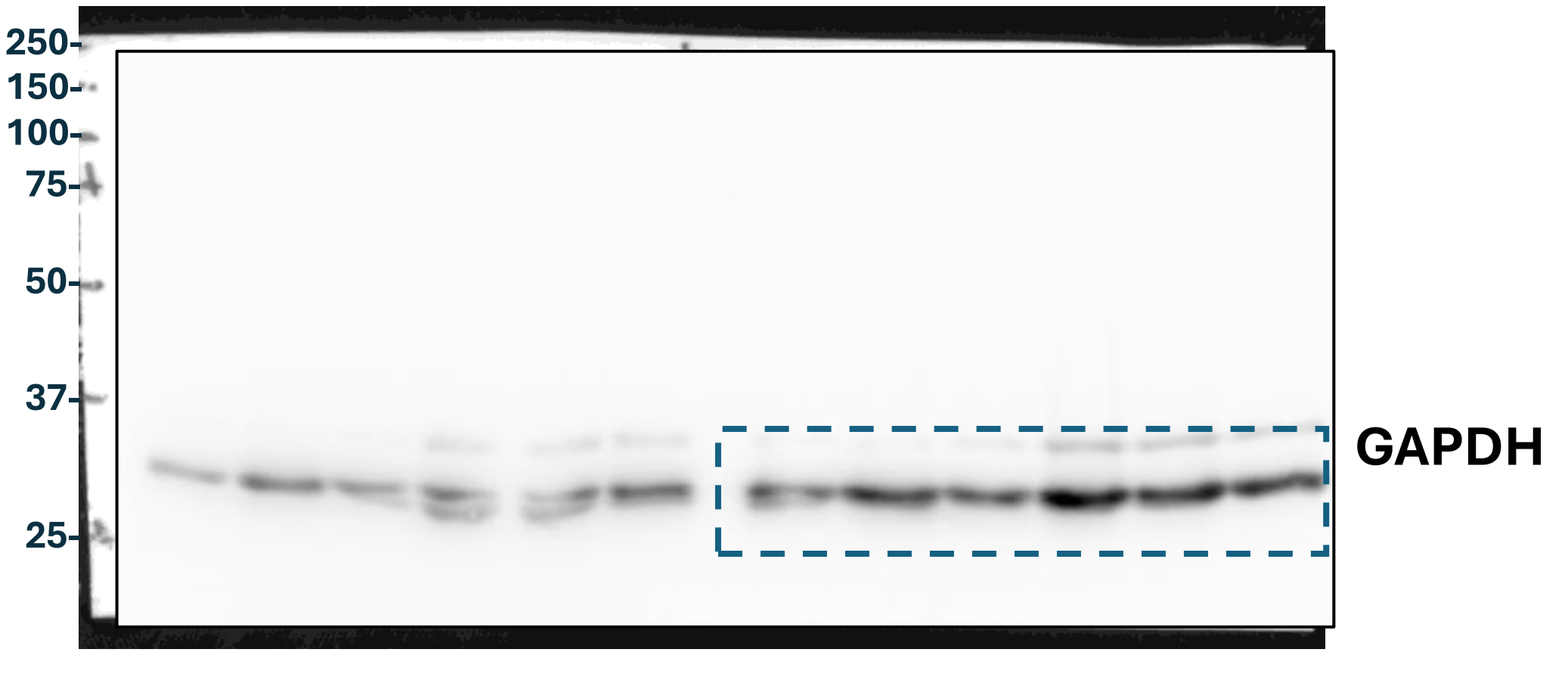

Figure 5F

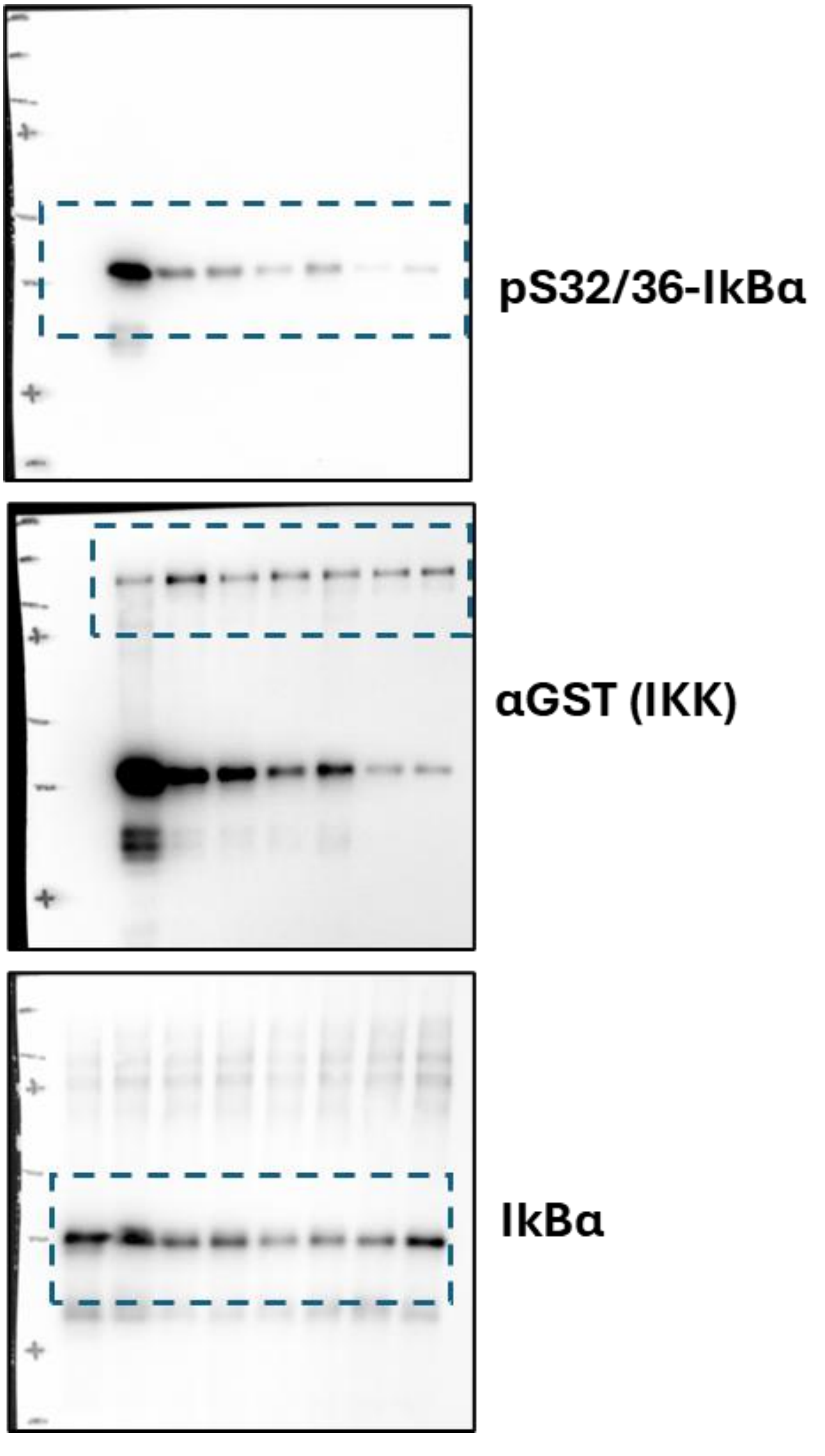

Suppl. Figure S4B

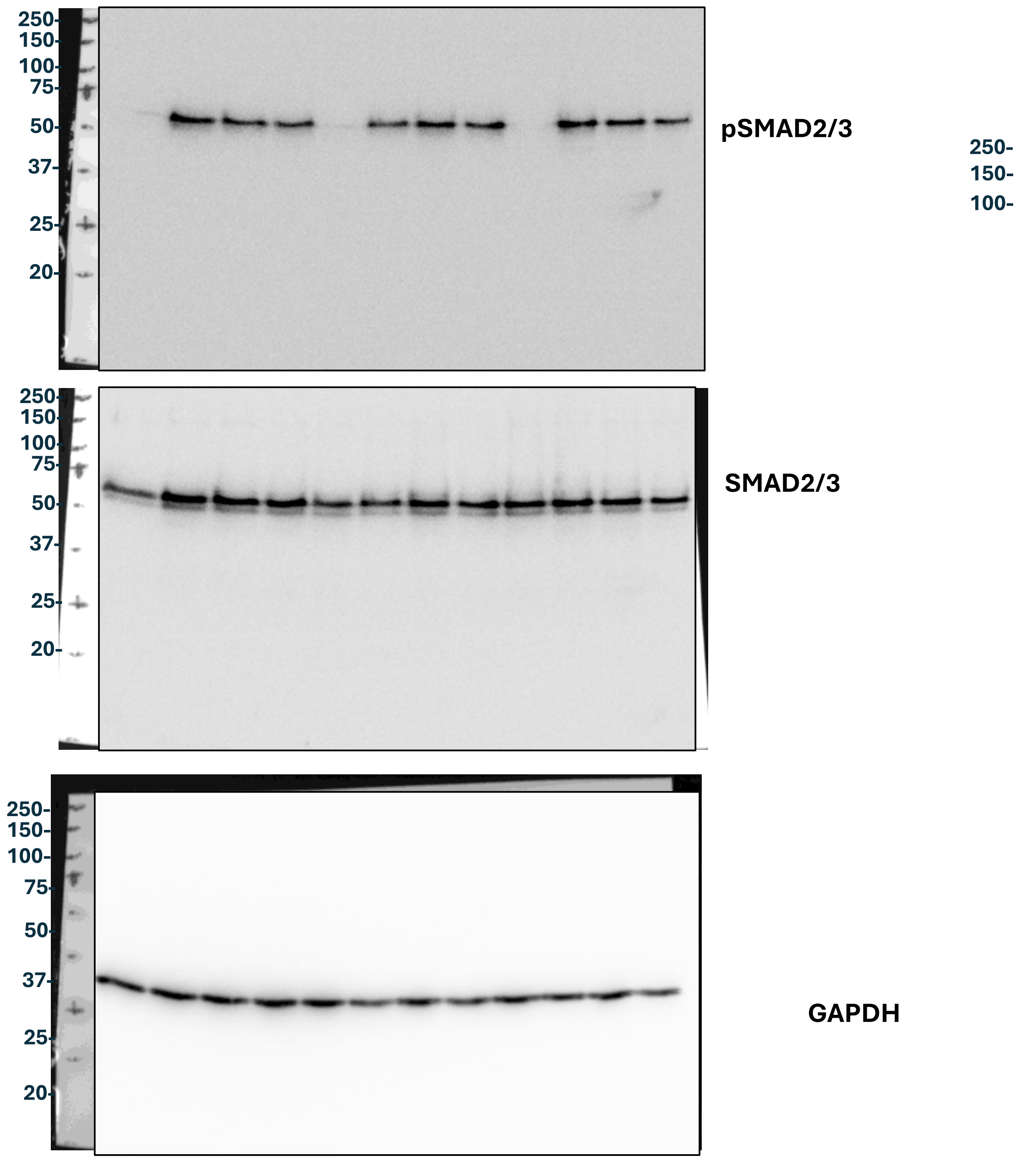

Suppl. Figure S4E

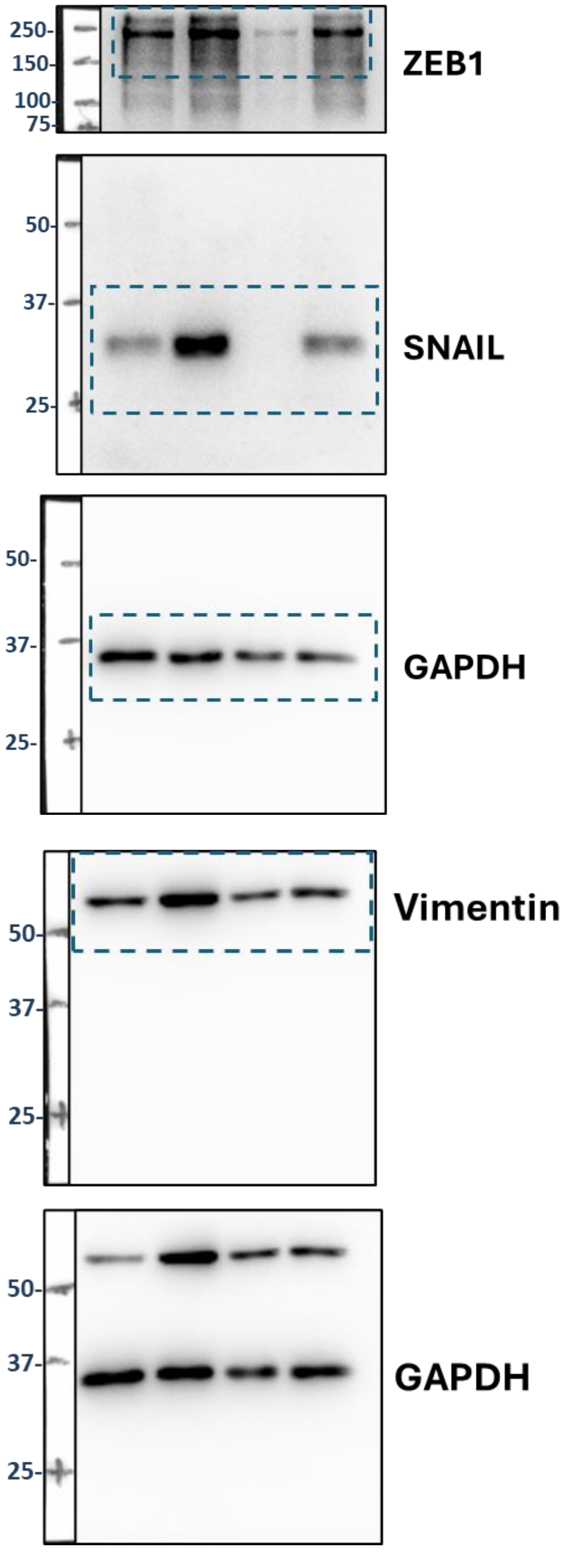

Supplement: Supplementary file 4 [file mmc5.pdf]
